# Effects of Taro (*Colocasia esculenta*) Water-Soluble Non-Starch Polysaccharide, *Lactobacillus* *acidophilus*, *Bifidobacterium breve*, *Bifidobacterium infantis*, and Their Synbiotic Mixtures on Pro-Inflammatory Cytokine Interleukin-8 Production

**DOI:** 10.3390/nu14102128

**Published:** 2022-05-19

**Authors:** Mylene Anwar, Sonya Mros, Michelle McConnell, Alaa El-Din A. Bekhit

**Affiliations:** 1 Department of Food Science, University of Otago, P.O. Box 56, Dunedin 9054, New Zealand; myleneanwar@cmu.edu.ph; 2 Department of Food Science, Central Mindanao University, University Town, Musuan, Maramag 8710, Bukidnon, Philippines; 3 Department of Microbiology and Immunology, University of Otago, P.O. Box 56, Dunedin 9054, New Zealand; sonya.mros@otago.ac.nz (S.M.); michelleandstewartmcconnell@gmail.com (M.M.)

**Keywords:** taro (*Colocasia esculenta*), water-soluble non-starch polysaccharide, probiotics, synbiotic, interleukin 8, necrotising enterocolitis

## Abstract

In the past decades, the regulation of pro-inflammatory cytokine production, including interleukin-8 (IL-8), has been the goal of many targeted therapeutic interventions for Necrotising enterocolitis (NEC), a gastrointestinal disease commonly associated with a very low birth weight in preterm infants. In this study, the ability to regulate the production of IL-8 of the water-soluble non-starch polysaccharide (WS-NSP) from taro corm (Tc-WS-NSP) extracted using a conventional (CE) or improved conventional (ICE) extraction method, of the probiotics *Lactobacillus acidophilus*, *Bifidobacterium breve*, and *Bifidobacterium infantis*, and their synbiotic mixtures were evaluated. The TNF-α stimulated HT-29 cells were incubated with undigested or digested Tc-WS-NSPs (CE or ICE), probiotics, and their synbiotic mixtures with *Klebsiella oxytoca*, an NEC-positive-associated pathogen. Overall, the synbiotic mixtures of digested Tc-WS-NSP-ICE and high bacterial concentrations of *L. acidophilus* (5.57 × 10^9^), *B. breve* (2.7 × 10^8^ CFU/mL), and *B. infantis* (1.53 × 10^8^) demonstrated higher (42.0%, 45.0%, 43.1%, respectively) ability to downregulate IL-8 compared to the sole use of Tc-WS-NSPs (24.5%), or the probiotics *L. acidophilus* (32.3%), *B. breve* (37.8%), or *B. infantis* (33.1%). The ability demonstrated by the Tc-WS-NSPs, the probiotics, and their synbiotics mixtures to downregulate IL-8 production in the presence of an NEC-positive-associated pathogen may be useful in the development of novel prophylactic agents against NEC.

## 1. Introduction

Necrotising enterocolitis (NEC) is a progressive disease of the neonatal intestine characterized by inflammation of the gut wall that may advance to necrosis and gut perforation [1]. It typically affects very low birth weight (≤1500 g), preterm infants (born less than 37 weeks) who account for the majority (70% to 90%) of cases [2]. It is associated with significant morbidity due to complications associated with the disease [3] and remains a leading cause of mortality of premature infants in the neonatal intensive care unit [4]. Despite medical interventions involving discontinuation of enteral feeds, administration of antibiotics, supportive care [5], and surgical treatment [6], infants who have the disease may die or suffer from potential long-term health effects including short-bowel syndrome, poor growth, post-surgical complications, and neurodevelopmental challenges [7,8].

Pathogenic bacteria have been implicated in the pathogenesis of the disease based on clinical cases of NEC [9]. However, to date, there is no strong evidence of a specific pathogen linked to NEC [10]. It is believed that rather than a direct infection by a specific pathogen, NEC develops as a result of the adherence of the pathogenic microorganisms or their toxin on the intestinal wall that triggers an exaggerated inflammatory response characterized by the production of a high amount of cytokines, particularly IL-8, by the enterocytes [11]. The unregulated production of high amounts of pro-inflammatory cytokines can initiate the inflammation process leading to the development of NEC [12]. The excessive IL-8 production by human foetal intestinal cell line after inflammatory stimulation has helped in part to explain the occurrence and development of NEC in premature infants [13] making IL-8 a good response biomarker to be studied. Thus, most of the interventions to prevent, or delay the progress of NEC have been aimed toward the regulation of pro-inflammatory cytokine production and enhancing the infant’s nutritional status for proper growth and development [14].

The use of prebiotics [15], probiotics [16,17,18], and synbiotics [19] as prophylactic agents and nutritional intervention to protect an infant from developing NEC has been explored recently. A number of studies have investigated the use of prophylactic agents such as inulin [20], galactooligosaccharides (GOS), fructooligosaccharides (FOS) [21], *Lactobacillus* and *Bifidobacterium* spp. [19,22], probiotic mixture of *L. acidophilus* and *B. infantis* [23], and synbiotic mixtures of inulin and *Bifidobacterium lactis* [20] and FOS and *L. acidophilus*, *Bifidobacterium longum*, *Bifidobacterium bifidum*, *Streptococcus thermophiles* [24,25,26] which were reported to reduce incidence and severity of NEC at various levels. These prophylactic agents were found to regulate the production of pro-inflammatory cytokines, including IL-8, which is one of the mechanisms believed to be beneficial in reducing the incidence and severity of NEC [15,16,27]. However, due to the variability of the reported efficacy of the various prophylactic agents against NEC and the need to further investigate appropriate prebiotic and probiotic dosage and synbiotic mixture combinations [28], more scientific investigations are required to explore the use of new prebiotic materials, efficient probiotics and synbiotic mixtures that can be useful as a prophylactic agent for NEC. Our previous study [29] demonstrated that taro (*Colocasia esculenta*), a tuberous root crop containing WS-NSP that can downregulate the production of IL-8 produced by tumor necrosis factor alpha (TNF-α) stimulated HT-29 cells, a gut epithelial cell line. The WS-NSP of taro corm (Tc-WS-NSP), unlike its starch, is considered a minor component [30]. It only accounts for 3.02 to 18.99% of the dry weight basis of the corm depending on the variety [31]. The Tc-WS-NSP is often not recovered during starch production or in the processing of taro-based products and is largely wasted by the taro industry [32]. Nonetheless, it is known to be a by-product of value due to its varied uses in food [33,34] and pharmaceutical [35] applications. It also exhibits a number of biological activities including antidiabetic potential [36], anti-oxidative [31], antimetastatic [37], anti-inflammatory, and immunomodulatory [38,39,40] activities. In addition, it can support the growth of *L. acidophilus*, *B. breve*, and *B. infantis* [29], which are probiotics capable of regulating the production of pro-inflammatory cytokine IL-8. These novel findings on the potential of Tc-WS-NSP, solely or in combination with probiotics suggest its usefulness as a prophylactic agent against intestinal diseases such as NEC. This study is the first to report the use of WS-NSP extracted from taro, and its synbiotic mixtures with the probiotics *L. acidophilus*, *B. breve*, and *B. infantis* as potential prophylactic agents against NEC. The potential of Tc-WS-NSP, the probiotics, and their synbiotic mixtures as prophylactic agents against NEC is evaluated based on their ability to regulate the production of the pro-inflammatory IL-8 by TNF-α stimulated HT-29 cells in the presence of an NEC-positive associated pathogen, *K. oxytoca*.

## 2. Materials and Methods

### 2.1. Materials

Fresh taro corms (*Colocasia esculenta*, pink cultivar from Fiji) of approximately 971.3 ± 17.8 g/corm were purchased from a local supermarket (Dunedin, Otago, New Zealand). Human colorectal adenocarcinoma epithelial cell line (HT-29, ATCC^®^, HTB-38™) and pure cultures of *L. acidophilus* (Infloran^®^), *B. infantis* (Infloran^®^), *B. breve* (Moringa^®^ M-16V), NEC-positive associated bacterium, *K. oxytoca* (clinical isolate from infant faecal sample, NICU, Dunedin Hospital, Dunedin, New Zealand), and non-pathogenic *Escherichia coli* (ATCC^®^ 25922™, Serotype O6, non-verotoxin producer, quality control strain (Biosafety level 1); New Zealand Reference Culture Collection: Medical Section (NZRM) 916) were provided by the Department of Microbiology and Immunology, University of Otago, New Zealand. Bacterial culture media including de Man, Rogosa, and Sharpe (MRS), Trypticase soy broth, and agar were purchased from Difco Laboratories Inc. (Difco™ dehydrated Culture Media, Franklin Lakes, NJ, USA). Anaerobic packs (AnaeroPack™) were purchased from Mitsubishi Gas Chemical Inc. (Ngaio Diagnostics, Nelson, New Zealand). Membrane filters (MF-Millipore™ membrane filter, 0.22 µm pore size) were purchased from Merck (Merck, Auckland, New Zealand). ELISA plates (MaxiSorp™, NUNC™) and tissue culture flasks (NUNC™) were purchased from Thermo Fischer Scientific (Auckland, New Zealand). The IL-8 ELISA test kit (OptEIA™) was purchased from BD Biosciences (San Diego, CA, USA). Ultrafilter concentrators (Vivaspin 200™, 30,000 MWCO, Cytiva) and Methylthiazolyldiphenyl-tetrazolium bromide (MTT) were purchased from Life Technologies (Thermo Fischer Scientific, North Shore, New Zealand). Dulbecco’s modified eagle medium (DMEM), penicillin (10,000 U/mL), streptomycin (10,000 μg/mL), TrypLE™ express, and foetal bovine serum (FBS) were purchased from Gibco^®^ (Thermo Fischer Scientific, North Shore, New Zealand). Disodium hydrogen phosphate, sodium chloride, potassium chloride, and potassium dihydrogen phosphate used in the preparation of phosphate-buffered saline (PBS) were purchased from BDH^®^ Reagents (VWR International, Rochester, NY, USA). The α-amylase (*Aspergillus oryzae* α-amylase, ~30 U/mg), pepsin (powdered porcine gastric mucosa pepsin, ≥250 U/mg), pancreatin (porcine pancreas pancreatin), and other chemicals and reagents were purchased from Sigma-Aldrich (Merck KGaA, Darmstadt, Germany) and were of analytical grade.

### 2.2. Methods

#### 2.2.1. Extraction of Taro Water-Soluble Non-Starch Polysaccharides (Tc-WS-NSP)

The extraction of Tc-WS-NSP was undertaken using a conventional extraction (CE) and an improved conventional extraction (ICE) method utilizing freeze-thaw [41]. In both CE and ICE, distilled water (pH 6.0, 4 °C) was added to the taro corm slices at a ratio of 1:1 (*v*/*w*). For CE, the Tc-WS-NSP was extracted using the method of [42] modified with the use of ultrafiltration [43]. Briefly, the taro-water mixture was homogenized (1 min) using an industrial blender (Conair™, Waring™, Thermo Fischer Scientific, Austin, TX, USA), then filtered (100 μm, Tyler Mesh Sieve, Mentor, OH, USA) before the extract was centrifuged (Beckman Coulter Life Sciences, Indianapolis, IN, USA) at 11,180× *g* for 10 min at 4 °C. The supernatant was collected and concentrated under vacuum using a rotary evaporator (Rotavap™, BÜCHI Labortecknik AG, Flawil, Switzerland) at 40 °C. The concentrated extract was ultra-filtered using Vivaspin 200™ (30,000 MWCO) and centrifuged at 1789× *g* for 30 min at 4 °C. The filtrate was precipitated using ethanol (95%, *w*/*v*) at a ratio of 3:1 ethanol to extract (*v*/*v*) for 8 h. The precipitates were collected after centrifugation at 11,180× *g* for 10 min at 4 °C and washed with ethanol (95%, 5 mL), followed by acetone (5 mL) for three times each. The solvents were evaporated using N_2_ gas. The Tc-WS-NSP extracts were stored in a desiccator containing silica gel to allow partial drying for 24 h. Partially dried Tc-WS-NSP extracts were frozen overnight at −30 °C and freeze-dried (Labconco™ Freeze-dryer, Kansas City, MO, USA). For ICE, the taro-water mixture was frozen in a −30 °C chest freezer (GE^®^ Appliances, Auckland, New Zealand) for 12 h and then thawed at 25 °C for 4 h. The thawed extract was filtered (100 μm Tyler Mesh Sieve, Mentor, OH, USA) and centrifuged at 11,180× *g* for 10 min at 4 °C. The supernatant was collected, concentrated under vacuum, ultrafiltered, precipitated, and freeze-dried using similar conditions of the CE as described above. The freeze-dried Tc-WS-NSP-CE and Tc-WS-NSP-ICE samples were stored in airtight vials at −20 °C for further analyses.

#### 2.2.2. Digestion of Tc-WS-NSP

The extracted Tc-WS-NSP-CE and Tc-WS-NSP-ICE were digested using a three-stage (salivary, gastric, and intestinal) simulated in vitro digestion process following the methods of [44,45] with modification of initial sample concentration. A 1% (*w*/*v*) sample solution was used instead of 2% (*w*/*v*) [45] due to the high viscosity of Tc-WS-NSP samples at high (>1%, *w*/*v*) concentration. For salivary digestion, 20 mL of Tc-WS-NSP solutions (1%, *w*/*v*) was added to 6 mL of artificial saliva medium composed of 89.6 g/L KCl, 20.0 g/L KSCN, 88.8 g/L NaH_2_PO_4_, 57.0 g/L Na_2_SO_4_, 175.3 g/L NaCl, 84.7 g/L NaHCO_3_, 2.0 g/L urea and 290 mg α-amylase. The pH of the mixture was adjusted to 6.8 using 0.1 M HCl prior to the addition of 40 mL distilled water. The mixtures were incubated in a temperature-controlled shaker/incubator (Ratek Instruments, Victoria, Australia) at 37 °C for 5 min. Gastric digestion followed beginning with adjusting the pH of the mixtures to 2.0 using 2 M HCl. After pH adjustment, pepsin (600 μL in 0.1 M HCl) was added to initiate the gastric digestion process. The mixtures were again incubated in a temperature-controlled shaker/incubator at 37 °C for 2 h. For intestinal digestion, the pH of the mixtures was adjusted to 6.5 using 0.5 M NaHCO_3_ and 5 mL of pancreatin (8 mg/mL) and a bile salts (50 mg/mL) mixture (1:1, *v*/*v*) was added to the solution. The mixtures were again incubated in a temperature-controlled shaker/incubator at 37 °C for a further 2 h. The digested Tc-WS-NSP samples were recovered by adjusting the pH of the mixtures to neutral (pH 7.0) using 1 M HCl prior to centrifugation at 3000 rpm at 4 °C for 10 min. The supernatants were precipitated with three times the volume of ethanol (95%, *v*/*v*). The collection of the digested Tc-WS-NSP samples was performed by centrifugation (Beckman CPR centrifuge, Beckman Coulter Life Sciences, Lakeview, Indianapolis, IN, USA) at 10,000 rpm for 10 min at 4 °C. The digested Tc-WS-NSP samples were washed with ethanol (95%, *v*/*v*, 5 mL) followed by acetone (5 mL) for three times each. Solvents were allowed to evaporate using N_2_ gas and solvent-free digested Tc-WS-NSP samples were stored in a desiccator containing silica gels to allow complete drying. Dried digested Tc-WS-NSP samples were pulverized, sieved (100 μm), stored in airtight vials, and stored at −20 °C for further analyses.

#### 2.2.3. Culture Media and Bacterial Culture Preparations

All culture media were prepared according to the manufacturer’s instructions and sterilized at 121 °C, 15 psi (103 kPa) for 15 min. The media were allowed to solidify in 90 × 15 mm polystyrene petri dishes (LabServe^®^, Thermo Fischer, Auckland, New Zealand).

For the bacterial culture preparation of each of the probiotic *L. acidophilus*, *B. breve*, and *B. infantis*, frozen (−80 °C) pure cultures of each of the probiotics were revived by allowing them to grow in MRS broth and subsequently on MRS agar. The probiotics were sub-cultured twice before the experimental tests to allow maximum recovery from the freezing-thawing process. A 1% (*v*/*v*) inoculum was sub-cultured into 20 mL pre-warmed (37 °C) sterile MRS broth and incubated in an anaerobic container system containing an anaerobic pack (AnaeroPack™, Mitsubishi Gas Chemical Inc., Ngaio Diagnostics, Nelson, New Zealand) as an oxygen absorber and CO_2_ generator at 37 °C for 48 h. After 48 h incubation, a loopful of the pure cultures were streaked into sterile MRS agar and incubated in an anaerobic container system (AnaeroPack™ 7.0 L Rectangular Jar, Thermo Fischer Scientific, North Shore, New Zealand) containing anaerobic packs at 37 °C for 48 h. The MRS broth and the solidified sterile MRS agar were pre-incubated in the anaerobic container system prior to use. For *K. oxytoca* and the non-pathogenic *E. coli*, the bacterial cultures were grown in 20 mL of sterile tryptic soy broth (TSB) for 24 h at 37 °C. The bacterial cultures used were also sub-cultured twice in tryptic soy agar (TSA) prior to its use for the experiment. A standard curve for each of the probiotics, *K. oxytoca*, and the non-pathogenic *E. coli* was established based on the culture medium’s optical density (OD_600_) against bacterial concentration (CFU/mL) using serial dilutions of 10^−1^ to 10^−8^ with sterile PBS (pH 7.2) prepared by dissolving 8.0 g NaCl, 1.16 g Na_2_HPO_4_, and 0.2 g KCl in 1 L of Milli Q water as diluent. Approximately 10 µL of the probiotics, *K. oxytoca* and the non-pathogenic *E. coli* bacterial suspensions were inoculated using a drop plate technique in MRS and Tryptic soy agar, respectively. From the standard curve, a bacterial load of 3.1 × 10^6^ CFU/mL (OD_600_ = 0.1) and 5.6 × 10^9^ CFU/mL (OD_600_ = 0.2) for *L. acidophilus*, 3.7 × 10^5^ CFU/mL (OD_600_ = 0.1) and 2.7 × 10^8^ CFU/mL (OD_600_ = 0.2) for *B. breve*, 4.6 × 10^5^ CFU/mL (OD_600_ = 0.1) and 1.5 × 10^8^ CFU/mL (OD_600_ = 0.2) for *B. infantis*, 4.0 × 10^6^ CFU/mL (OD_600_ = 0.1) and 2.1 × 10^7^ CFU/mL (OD_600_ = 0.2) for *K. oxytoca*, and 4.3 × 10^6^ CFU/mL (OD_600_ = 0.1) and 1.3 × 10^7^ CFU/mL (OD_600_ = 0.2) for the non-pathogenic *E. coli* were used to represent low and high bacterial concentrations. Furthermore, the bacterial suspensions of *K. oxytoca* and the non-pathogenic *E. coli* were subjected to heat-kill treatment at 80 °C for 45 min in a temperature-controlled water bath (Grant Instruments, heated circulating baths, Cambridge, UK) with agitation to inactivate the bacterial cells. To evaluate the efficiency of the heat-killing process, 10 μL of the heat-treated *K. oxytoca* and the non-pathogenic *E. coli* bacterial suspensions were cultured on Tryptic soy agar (TSA) using drop plate technique and incubated aerobically at 37 °C for 24 h to 48 h. The bacterial cells of each of the probiotics, *K. oxytoca*, and the non-pathogenic *E. coli* were collected by centrifugation (Biofuge 13, Heraeus, Sepatech, Thermoscientific™, Auckland, New Zealand) at 3000× *g* for 5 min. The bacterial pellets were washed twice with pre-warmed (37 °C) sterile PBS and re-collected by centrifugation. The preparations and incubation of the probiotics were performed in an anaerobic workstation (Whitley A35 Anaerobic workstation, Don Whitley Scientific, West Yorkshire, UK).

#### 2.2.4. Cell Culture Preparations

The human colorectal adenocarcinoma epithelial cell line (HT-29, ATCC^®^, HTB-38™) was grown in tissue culture flasks (Nunc™, Thermo Scientific™, Bartlett, IL, USA) containing the complete media composed of DMEM, 10% (*v*/*v*) FBS, 1% (*v*/*v*) penicillin (10,000 U/mL) and streptomycin (10,000 μg/mL). The cells were incubated at 37 °C with 95% humidity and 5% CO_2_ (Forma™, Steri-Cycle™, CO_2_ incubator, Thermo Fischer Scientific, North Shore, New Zealand). Following incubation (48 h), the cells were harvested and 100 μL per well of approximately 3 × 10^5^ cells/mL in complete medium were seeded into 96 well plates. The cells were incubated as described above. When wells reached 80% cell confluence, the culture media was discarded, and cells were washed twice with pre-warmed (37 °C) PBS and used in the subsequent experiments.

#### 2.2.5. Cytotoxicity Evaluation Using 3-(4,5-Dimethylthiazol-2-yl)-2,5-diphenyltetrazolium Bromide (MTT) Assay

The washed cells as described above were treated with 200 μL of filtered (0.2 µm filter, MF-Millipore™, Merck, Auckland, New Zealand) sterilized undigested or digested Tc-WS-NSP-CE or Tc-WS-NSP-ICE samples (1%, *v*/*v* of 1 mg/mL), each of the live probiotics in pellets of known bacterial concentrations, synbiotic mixtures, non-heat-killed and heat-killed *K. oxytoca*, and the non-pathogenic *E. coli* in pellets of known bacterial concentrations dissolved or dispersed in pre-warmed (37 °C) complete cell culture media (without antibiotic) with the stimulant (10 ng/mL of TNF-α). The TNF-α was used as the stimulant since it exhibited the greatest stimulatory effects indicated by the highest concentration of IL-8 (3832 pg/mL) than IL-1β (2116 pg/mL) and LPS (1612 pg/mL) based on the screening of inflammatory stimulus conducted for 24 h incubation period [46]. Untreated cells served as the control sample. The plates were incubated for 24 h at 37 °C with 95% humidity and 5% CO_2_ (Thermo Fischer Scientific, Forma™, Steri-Cycle™, CO_2_ incubator, North Shore, New Zealand). Following incubation, an MTT assay was conducted [47].

#### 2.2.6. Incubation of Undigested or Digested Tc-WS-NSPs, the Probiotics, and Their Synbiotic Mixtures on IL-8 Production by TNF-α Stimulated HT-29 Cells in the Presence of NEC-Positive Associated Pathogenic Bacterium *K. oxytoca* and Non-Pathogenic *E. coli*

The washed cells as described above were treated with 200 μL of complete media (without antibiotic) with the inflammatory stimulus TNF-α (10 ng/mL). The stimulated cells were incubated with either the filtered sterilised undigested or digested Tc-WS-NSP-CE or Tc-WS-NSP-ICE (1%, *v*/*v* of 1 mg/mL), each of the live probiotics, and the synbiotic mixtures with the heat-killed *K. oxytoca* or the non-pathogenic *E. coli* in bacterial pellets of known bacterial concentrations (CFU/mL). Untreated TNF-α stimulated cells and cells incubated only with *K. oxytoca* and the non-pathogenic *E. coli* served as control and reference samples, respectively. The plates were incubated (Thermo Fischer Scientific, Forma™, Steri-Cycle™, CO_2_ incubator, North Shore, New Zealand) for 24 h at 37 °C with 95% humidity and 5% CO_2_.

#### 2.2.7. IL-8 Quantification in Cell Culture Supernatants Using Enzyme-Linked Immunosorbent Assay (ELISA)

Following the 24 h incubation period, the IL-8 produced by the TNF-α stimulated HT-29 cells in the cell culture supernatants were analysed using ELISA (IL-8 ELISA test kit, OptEIA™, BD Biosciences, San Diego, CA, USA) according to the manufacturer’s protocol as described previously [29].

#### 2.2.8. Statistical Analysis

Statistical analysis and graphical presentation were performed using Minitab^®^ Software Version 16 (Minitab Inc., State College, PA, USA). ANOVA (general linear model) followed by Tukey’s test were used to evaluate the significant (*p* *≤* 0.05) differences among treatments. The data were expressed as means of three independent experiments (*n =* 3) ± standard deviation.

## 3. Results

### 3.1. Cytotoxicity of Live K. oxytoca and Non-Pathogenic E. coli on TNF-α Stimulated HT-29 Cells

**Figure 1:**
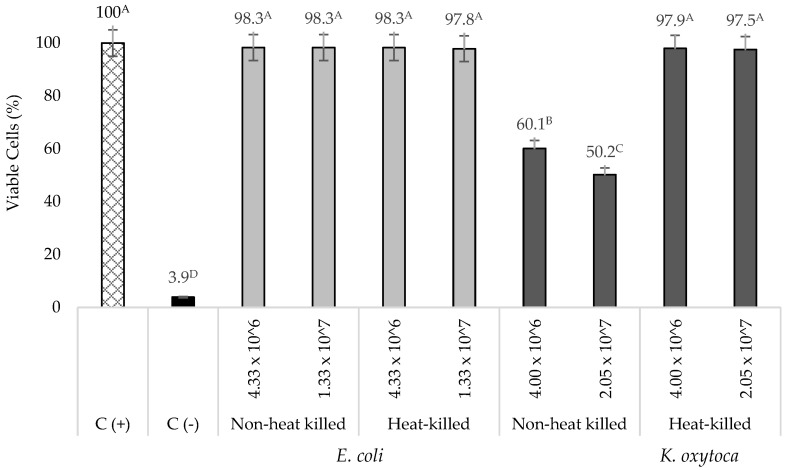
Viable TNF-α-stimulated HT-29 cells (%) incubated with non-heat killed and heat killed *K. oxytoca* and *E. coli* at different bacterial concentrations (CFU/mL). Bars that do not share the same letter are significantly (*p* *≤* 0.05) different (ANOVA with Tukey pairwise comparison). Grouping information for significant differences: A–D, viable cells among treatments compared to control samples.

The incubation (24 h) of low (4.0 × 10^6^ CFU/mL) and high (2.1 × 10^7^ CFU/mL) bacterial concentrations of live *K. oxytoca* in TNF-α stimulated HT-29 cells resulted in a 39.9 ± 3.8 and 49.8 ± 3.2% decrease in viable cells, respectively (Figure 1). When heat-killed, *K. oxytoca* did not exhibit a cytotoxic effect on the TNF-α stimulated HT-29 cells as evident by the high percentage (>95%) of viable cells. A number of in vitro studies have shown that non-heat killed K. oxytoca causes cell death to various cell lines including Hep-2, HeLa, Vero, and HT-29 cells [48,49,50]. This effect is attributed to its ability to produce the cytotoxins tilivalline and tilimycin that can induce cell death and are also reported to be responsible for the organism’s pathogenesis [51]. Thus, a heat-killed *K. oxytoca* was used in this study. On the other hand, non-heat killed and heat-killed non-pathogenic *E. coli* did not show significant cytotoxic effects on TNF-α stimulated HT-29 cells (Figure 1). However, for comparison purposes, a heat-killed non-pathogenic *E. coli* was also used to further evaluate the effects of NEC-positive associated bacterium (*K. oxytoca*) and negative control bacterium (non-pathogenic *E. coli*) on IL-8 production by TNF-α stimulated HT-29 cells incubated with undigested or digested Tc-WS-NSPs and probiotics.

### 3.2. Cytotoxicity of Undigested or Digested Tc-WS-NSPs, Live Probiotics, and Their Synbiotic Mixtures with Heat-Killed K. oxytoca or E. coli on TNF-α Stimulated HT-29 Cells

The results of the cytotoxicity evaluation of the undigested or digested Tc-WS-NSP-CE or Tc-WS-NSP-ICE, each of the live probiotics, and their synbiotic mixtures in the presence of heat-killed *K. oxytoca* or the non-pathogenic *E. coli* showed that there was no significant (*p* *≥* 0.05) difference in the number of viable cells between the untreated and treated cells (Supplementary Materials Figures S1–S5). Natural polysaccharides, including WS-NSPs, are generally known to be non-toxic or have low cytotoxicity in mammalian cells [52,53]. Regarding the cytotoxicity of Tc-WS-NSP, our previous study [40] demonstrated the lack of or minimal cytotoxicity of both the undigested or digested Tc-WS-NSPs extracted using the CE and ICE methods in HT-29 cells in a concentration range of 0.5 to 7.5 mg/mL. Regarding the cytotoxicity of the probiotics, a number of studies on *L. acidophilus*, *B. breve*, and *B. infantis* [54,55,56,57] have shown that these probiotics have no cytotoxic effects on cells including HT-29 cell culture. This has been established based on the safety profile of various clinical trials using these probiotics [58,59,60,61]. Since the non-heat killed probiotics in this study did not show cytotoxic effects on the cell culture, viable bacterial cultures were used in the succeeding experiments. The finding suggests that the Tc-WS-NSP samples, each of the probiotics, and their synbiotic mixtures with heat-killed *K. oxytoca* or *E. coli* do not exert or have a very minimal cytotoxic effect on HT-29 cells. The results are favourable as it allows further investigation of the potential of the Tc-WS-NSP samples, the probiotics, and their synbiotic mixtures to regulate IL-8 production by the TNF-α stimulated HT-29 cells in the presence of an NEC-positive associated bacterium, *K. oxytoca* or a non-pathogenic bacterial isolate (i.e., *E. coli*).

### 3.3. IL-8 Production by TNF-α Stimulated HT-29 Cells upon Incubation with Heat-Killed NEC-Positive Associated Bacterium K. oxytoca or Non-Pathogenic E. coli

The incubation of low (4.0 × 10^6^ CFU/mL) and high (2.1 × 10^7^ CFU/mL) bacterial concentrations of *K. oxytoca* resulted in an increase of 33.8 ± 2.8% (Supplementary Materials Figures S6 and S7), 44.0 ± 3.9% (Supplementary Materials Figures S8–S10), 33.8 ± 2.0% (Supplementary Figures S6 and S7), and 45.8 ± 1.4% (Supplementary Materials Figures S8–S10) in IL-8 production by the stimulated HT-29 cells, respectively. On the other hand, the incubation of *E. coli* at bacterial concentrations of 4.3 × 10^6^ and 1.3 × 10^7^ CFU/mL only caused a 2.2 ± 1.6% (Supplementary Materials Figures S6 and S7), 3.3 ± 1.4% (Supplementary Materials Figures S8–S10), 3.0 ± 1.0% (Supplementary Materials Figures S6 and S7), and 3.2 ± 2.1% (Supplementary Materials Figures S8–S10) increase in IL-8 concentration. In general, the increase in IL-8 concentration with heat-killed NEC-positive associated bacterium *K. oxytoca* was significantly (*p* *≤* 0.05) higher than the increase in IL-8 concentration upon incubation with the non-pathogenic *E. coli*.

### 3.4. Effects of Undigested or Digested Tc-WS-NSPs on IL-8 Production by TNF-α Stimulated HT-29 Cells in the Presence of Heat-Killed NEC-Positive Associated Bacterium K. oxytoca or Non-Pathogenic E. coli

The increase in IL-8 concentration with the incubation of *K. oxytoca* and *E. coli* was downregulated with the incubation of undigested or digested Tc-WS-NSP-CE or Tc-WS-NSP-ICE to a varying extent. In cells with *K. oxytoca* and undigested or digested Tc-WS-NSP-CE or Tc-WS-NSP-ICE, IL-8 reductions of 16.8 ± 2.0 and 24.5 ± 3.4% were observed from the IL-8 concentrations of 5370.8 ± 137.6 pg/mL (4.0 × 10^6^ CFU/mL *K. oxytoca*) and 5367.1 ± 187.8 pg/mL (2.1 × 10^7^ CFU/mL *K. oxytoca*) compared to the control samples (Table 1). In cells with *E. coli*, a significantly (*p* *≤* 0.05) lower IL-8 reductions of 5.2 ± 2.0 and 17.1 ± 2.7% were observed in treatments incubated with undigested or digested Tc-WS-NSP-CE or Tc-WS-NSP-ICE from the IL-8 concentrations of 3634.7 ± 146.1 and 3665.3 ± 128.5 pg/mL of the control samples (Table 1).

**Figure 2:**
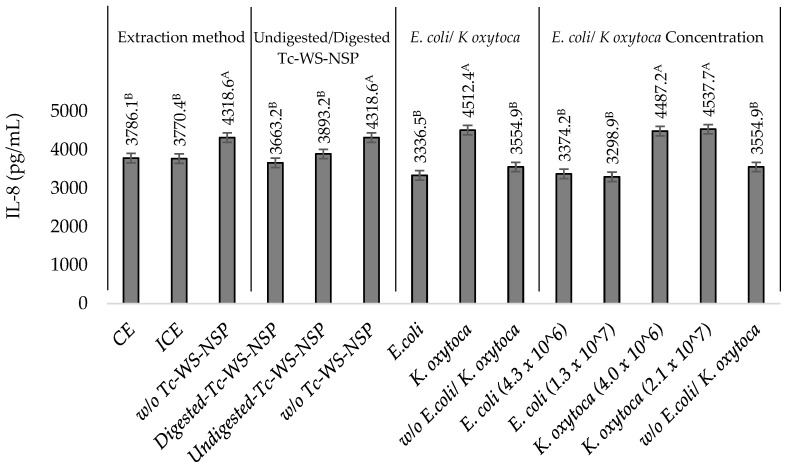
IL-8 produced by TNF-α stimulated HT-29 cells incubated with undigested or digested water-soluble non-starch polysaccharide from taro corm (Tc-WS-NSP) extracted using the conventional extraction (CE) and improved conventional extraction (ICE) methods. Bars that do not share the same letter are significantly (*p ≤* 0.05) different (ANOVA with Tukey pairwise comparison).

The digestion of the Tc-WS-NSP and the type of bacterial isolate (*K. oxytoca* or *E. coli*) significantly (*p* *≤* 0.05) affected the ability of the Tc-WS-NSP to downregulate IL-8 produced by the stimulated cells in the presence of *K. oxytoca* or *E. coli*, while the extraction method and bacterial concentrations of *K. oxytoca* and *E. coli* did not have significant (*p >* 0.05) effects. The influence of the digestion process was indicated by the higher reduction in IL-8 concentration upon incubation with digested Tc-WS-NSP than with incubating undigested Tc-WS-NSP (Figure 2). This observed difference in the ability of the undigested or digested Tc-WS-NSP to downregulate the production of IL-8 can be attributed to the dissimilarity in some important properties (i.e., viscosity) of the Tc-WS-NSP samples.

The down-regulation capacities of the undigested or digested Tc-WS-NSP-CE or Tc-WS-NSP-ICE were more apparent in cells incubated with *K. oxytoca* than with *E. coli* (Figure 2). An IL-8 reduction from 17.2 ± 3.0 to 24.5 ± 3.4% and 16.8 ± 2.0% to 21.8 ± 1.0% (Table 1) was obtained upon incubation of low (4.0 × 10^6^ CFU/mL) and high (2.1 × 10^7^ CFU/mL) bacterial concentrations of *K. oxytoca* with undigested or digested Tc-WS-NSP-CE or Tc-WS-NSP-ICE, respectively. On the other hand, incubation of low (4.3 × 10^6^ CFU/mL) and high (1.3 × 10^7^ CFU/mL) bacterial concentrations of *E. coli* with undigested or digested Tc-WS-NSP-CE or Tc-WS-NSP-ICE had an IL-8 reduction of only 5.2 ± 2.0 to 8.4 ± 3.0% and 8.1 ± 0.7 to 17.1 ± 2.7% (Table 1). As mentioned earlier, pathogenic microorganisms have the ability to significantly induce IL-8 production by the IECs as a response to infection than in the presence of a non-pathogenic microorganism.

### 3.5. Effects of the Probiotics L. acidophilus, B. breve, and B. infantis on IL-8 Production by TNF-α Stimulated HT-29 Cells in the Presence of Heat-Killed NEC-Positive Associated Bacterium K. oxytoca or Non-Pathogenic E. coli

**Figure 3:**
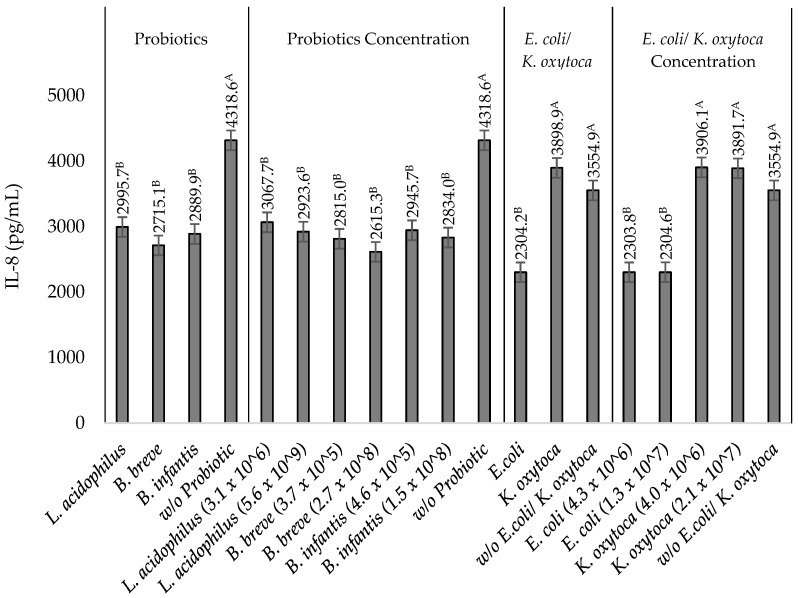
IL-8 produced by TNF-α stimulated HT-29 cells incubated with the probiotics *L. acidophilus*, *B. breve*, and *B. infantis* in the presence of non-pathogenic *E. coli* and NEC-positive associated pathogen *K. oxytoca*. Bars that do not share the same letter are significantly (*p* *≤* 0.05) different (ANOVA with Tukey pairwise comparison).

The incubation of the probiotics *L. acidophilus*, *B. breve*, and *B. infantis* using different bacterial concentrations in the presence of *K. oxytoca* and *E. coli* also resulted in a reduced IL-8 production by the stimulated HT-29 cells similar to incubating Tc-WS-NSP samples. In the presence of *K. oxytoca*, an IL-8 reduction of 28.5 ± 2.5 to 32.3 ± 1.5%, 31.7 ± 1.3 to 37.8 ± 1.9%, and 29.5 ± 1.0% to 33.1 ± 2.9% was obtained upon incubation with *L. acidophilus*, *B. breve* and *B. infantis*, respectively (Table 2). On the other hand, a significantly (*p* ≤ 0.05) lower IL-8 reduction was observed upon incubation of *L. acidophilus* (35.9 ± 5.1 to 40.9 ± 3.3%), *B. breve* (45.9 ± 4.1 to 50.2 ± 3.3%), and *B. infantis* (41.2 ± 3.9 to 44.3 ± 7.3%) in the presence of *E. coli* (Table 2). Furthermore, there is no significant (*p* ≥ 0.05) difference in IL-8 concentration among treatments of different bacterial isolates (*K. oxytoca* or *E. coli*) and in treatments with different bacterial concentrations of *K. oxytoca* or *E. coli* at low and high bacterial concentrations of each of the probiotics (Table 2). Regarding the IL-8 reduction among all treatments, the incubation of high bacterial concentrations of each of the probiotics *L. acidophilus* (5.6 × 10^9^ CFU/mL), *B. breve* (2.7 × 10^8^ CFU/mL), and *B. infantis* (4.6 × 10^8^ CFU/mL) with high bacterial concentrations of *K. oxytoca* (2.1 × 10^7^ CFU/mL) or *E. coli* (1.3 × 10^7^ CFU/mL) showed the largest IL-8 reductions of 32.3 ± 1.53 or 40.9 ± 3.3%, 37.8 ± 1.9 or 50.2 ± 3.3%, and 33.1 ± 2.9 or 44.3 ± 7.3%, respectively (Table 2). The bacterial concentration of *B. breve* has a significant (*p* ≤ 0.05) influence on its ability to downregulate IL-8 production, whereas the bacterial concentration of *L. acidophilus* and *B. infantis* did not show a significant (*p* ≥ 0.05) effect (Table 2). Among the probiotics, the high (2.7 × 10^8^ CFU/mL) bacterial concentration of *B. breve* demonstrated the highest (37.8 ± 1.9%) capacity to downregulate IL-8 production in cells incubated with high bacterial concentrations of *K. oxytoca* (2.1 × 10^7^ CFU/mL) (Figure 3). This finding suggests the strong potential of the probiotic *B. breve* as a prophylactic agent against NEC.

### 3.6. Effects of the Synbiotic Mixtures of the Undigested or Digested Tc-WS-NSPs and the Probiotics L. acidophilus, B. breve, and B. infantis on IL-8 Production by TNF-α Stimulated HT-29 Cells in the Presence of Heat-Killed NEC-Positive Associated Bacterium K. oxytoca or Non-Pathogenic E. coli

**Figure 4:**
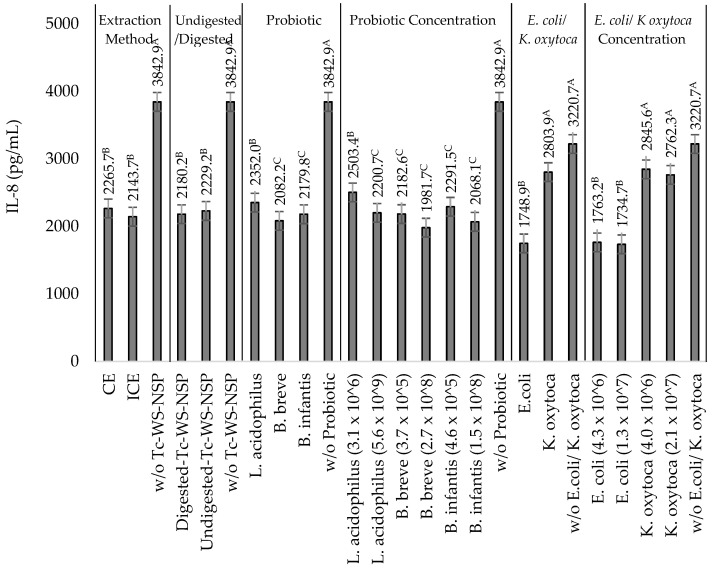
IL-8 produced by TNF-α stimulated HT-29 cells incubated with the synbiotic mixtures of water-soluble non-starch polysaccharide from taro corm (Tc-WS-NSP) extracted using the conventional extraction (CE) and improved conventional extraction (ICE) methods and each of the probiotics *L. acidophilus*, *B. breve*, and *B. infantis* in the presence of non-pathogenic *E. coli* and NEC-positive associated pathogen *K. oxytoca*. Bars that do not share the same letter are significantly (*p* *≤* 0.05) different (ANOVA with Tukey pairwise comparison).

Consistently, a decrease in IL-8 concentration was also observed upon incubation of the synbiotic mixtures of the undigested or digested Tc-WS-NSP samples and each of the probiotics at different bacterial concentrations in the presence of *K. oxytoca* or *E. coli*. In cells incubated with *K. oxytoca*, an IL-8 reduction of 32.5 ± 4.1 to 42.0 ± 2.5%, 38.2 ± 2.7 to 45.0 ± 2.5%, and 34.1 ± 2.3 to 43.1 ± 5.1% was obtained upon incubation with undigested or digested Tc-WS-NSP-CE or Tc-WS-NSP-ICE and the probiotics *L. acidophilus*, *B. breve* and *B. infantis*, respectively (Table 3, Table 4 and Table 5). On the other hand, a higher IL-8 reduction (40.5 ± 1.7 to 46.8 ± 2.9, 51.4 ± 2.6 to 58.1 ± 2.3%, and 49.2 ± 3.1 to 54.2 ± 3.6%) was observed in cells incubated with *E. coli* respective to the incubation of the probiotics *L. acidophilus*, *B. breve* and *B. infantis* with undigested or digested Tc-WS-NSP-CE or Tc-WS-NSP-ICE (Table 3, Table 4 and Table 5). The extraction method used to extract the Tc-WS-NSP did not show a significant (*p* ≥ 0.05) effect on the capacity of the synbiotic mixtures to downregulate IL-8 reduction in the presence of *K. oxytoca* or *E. coli*. However, the digestion process significantly (*p* ≤ 0.05) affects the capacity of the probiotics *L. acidophilus* and *B. infantis* in the synbiotic mixture to downregulate IL-8 production but not the probiotic *B. breve*. The incubation of digested Tc-WS-NSP, particularly digested Tc-WS-NSP-ICE, resulted in the probiotics *L. acidophilus* and *B. infantis* to have significantly higher (*p* ≤ 0.05) capacities to downregulate IL-8 production than the incubation of undigested Tc-WS-NSP (Figure 4). Among synbiotic mixtures with *L. acidophilus*, the highest IL-8 reductions of 46.3 ± 4.3 and 46.8 ± 2.9% or 41.3 ± 2.5 and 42.0 ± 2.5% were observed in cells incubated with high bacterial concentration (5.6 × 10^9^ CFU/mL) of L. acidophilus and digested Tc-WS-NSP-ICE with low and high bacterial concentrations of *E. coli* (4.3 × 10^6^ and 1.3 × 10^7^ CFU/mL) or *K. oxytoca* (4.0 × 10^6^ and 2.1 × 10^7^ CFU/mL), respectively (Figure 4). Similarly, the incubation of a high bacterial concentration of B. infantis (1.5 × 10^8^ CFU/mL) with digested Tc-WS-NSP-ICE had the largest IL-8 reductions of 54.2 ± 3.6 and 53.72 ± 2.30% or 40.9 ± 2.1 and 43.1 ± 5.1% in the presence of low and high bacterial concentrations of *E. coli* (4.3 × 10^6^ and 1.3 × 10^7^ CFU/mL) or *K. oxytoca* (4.0 × 10^6^ and 2.1 × 10^7^ CFU/mL), respectively (Figure 4). In synbiotic mixtures with *B. breve*, a higher IL-8 reduction of 55.2 ± 3.0 and 58.12 ± 2.33 or 43.15 ± 2.54 and 45.02 ± 2.50% compared with the synbiotic mixtures containing *L. acidophilus* and *B. infantis* were observed in cells incubated with high bacterial concentration (2.7 × 10^8^ CFU/ mL) of *B. breve* and digested Tc-WS-NSP with low and high bacterial concentrations of *E. coli* (4.3 × 10^6^ and 1.3 × 10^7^ CFU/mL) or *K. oxytoca* (4.0 × 10^6^ and 2.1 × 10^7^ CFU/mL), respectively (Figure 4).

## 4. Discussion

It is known that the presence of a high number of enteropathogenic bacteria or their toxins in the gastrointestinal tract can induce epithelial cell secretion of IL-8 and other pro-inflammatory cytokines in response to infection [62]. *K. oxytoca* and other bacterial species (i.e., *Cronobacter sakazakii* 50, *Cronobacter sakazakii* 2029, *Klebsiella pneumoniae* VIII 8, and *Eneterobacter cloacae* I 1) associated with the development of NEC have been reported to cause an increase in the production of IL-8 by HT-29 cells [63,64]. The induction of IL-8 by the pathogenic bacteria is part of the interaction of these bacteria with their host, and unless it is controlled appropriately, it can have damaging effects [65].

Owing to their viscosity, WS-NSPs are known to be capable of regulating (mostly delaying or slowing down) chemical or biological reactions [66]. The regulation is accomplished through various mechanisms such as limiting, blocking, or entrapping the reacting materials to minimize or hinder their associated chemical or biological reaction [67]. Our previous study [40] demonstrated that digested Tc-WS-NSP contained higher concentrations of the WS-NSP. It is indicated by the higher amount of total carbohydrates (CE: 96.4 ± 0.2 and IE: 98.4 ± 0.9 g/100 g) than the undigested Tc-WS-NSP (CE: 76.5 ± 0.9 and ICE: 86.1 ± 0.9 g/100 g). The higher amount of the WS-NSP in the digested Tc-WS-NSP contributed to its higher viscosity. The higher viscosity enhanced the capacity of the digested Tc-WS-NSP to downregulate IL-8 production than the undigested Tc-WS-NSP.

The incubation of digested Tc-WS-NSP in combination with either low or high bacterial concentrations of *K. oxytoca* or *E. coli* resulted in a lower IL-8 production by the stimulated cells compared to other treatment conditions. The downregulation of IL-8 production by the cells is attributed to the anti-adherence capacity or the ability of the prebiotics to interfere with the adhesion of the pathogenic bacteria in the IECs [68]. The interference with the adhesion is due to the adherence of the prebiotics to the binding sites (adhesins) of the pathogenic bacteria [69]. In fact, a number of studies [70,71,72] have already shown the ability of prebiotics mostly WS-NSPs to regulate the production of pro-inflammatory substances in the presence of pathogenic bacteria. The regulation of pro-inflammatory cytokine production has led to the prevention and the alleviation of various gastrointestinal diseases. For instance, the WS-NSP from blackcurrant (*Ribes nigrum*) seeds containing an arabinogalactan-protein (1 mg/mL) inhibited the adhesion of *Helicobacter pylori* to the human gastric epithelial AGS cells by about 25% [73]. The prebiotic GOS (16 mg/mL) also exhibited an adherence inhibition of 71% against *Cronobacter sakazakii* in a HEp-2 human cell line. *C. sakazakii* is another opportunistic pathogen associated with NEC in neonates [74]. The reduced IL-8 concentration upon incubation with Tc-WS-NSP suggests that there was interference with the adhesion of *K. oxytoca* to the TNF-α stimulated HT-29 cells. This interference by the Tc-WS-NSP resulted in cells producing less IL-8.

The probiotics studied have varying ability to downregulate IL-8 production by stimulated HT-29 cells in the presence of *K. oxytoca* or *E. coli*. The ability of the probiotics to downregulate IL-8 production is attributed to their ability to interfere with the adhesion of the pathogenic bacteria and their toxins into the IECs by competitive exclusion [75] and by coaggregation with the enteric pathogens [76]. The probiotics *L. acidophilus*, *B. breve* and *B. infantis* are known to be capable of adhering to IECs through their carbohydrate-protein complex binding components that can bind on the glycoconjugate receptors of the IECs [77,78]. Their adherence provides less opportunity for the pathogenic bacteria and their toxins to adhere to the cells. In effect, the cells produce fewer chemokines as a response for potential pathogen invasion [62]. This effect has been demonstrated in the study of O’Hara et al. [57]. The incubation of *B. infantis* in the presence of the pathogenic bacteria *Salmonella typhimurium* resulted in a 23.5% reduction in IL-8 produced by TNF-α stimulated HT-29 cells [57]. The difference observed in the capacity of *L. acidophilus*, *B. breve* and *B. infantis* to downregulate IL-8 produced by the stimulated HT-29 cells can be attributed to the variation of the bacterial species and strain used in this study. Species and strain-to-strain variability of the adherence capacity of probiotics to various cell lines (e.g., CaCo-2, HT-29, and T84) is attributed to some factors. These factors include the difference in the structural adhesives of the probiotics including the pili [79] and moonlighting proteins [80], the surface hydrophobicity where probiotics with higher surface hydrophobicity have better adhesion capacity [81] and the presence of other non-proteinaceous components such as the glycoconjugates’ exopolysaccharides present in the bacterial cell surface [82].

The incubation of a high bacterial concentration of each of the probiotics and digested Tc-WS-NSP resulted in the synbiotic mixture exhibiting greater ability to downregulate IL-8 production compared to other treatment combinations (Figure 4). This is attributed to the combined beneficial effect from the digested Tc-WS-NSP and the high bacterial concentration of the probiotics. Our previous study demonstrated the ability of the digested Tc-WS-NSP to support the growth of the probiotics *L. acidophilus*, *B. breve*, and *B. infantis* [29]. Thus, aside from the anti-adherence capacity of the digested Tc-WS-NSP against *K. oxytoca*, the digested Tc-WS-NSP may have also supported the growth and viability of the probiotics. In effect, more viable probiotics can interfere on the adherence of *K. oxytoca* on the stimulated HT-29 cells resulting in a reduced IL-8 production.

## 5. Conclusions

In conclusion, the undigested or digested Tc-WS-NSP-CE or Tc-WS-NSP-ICE, the probiotics *L. acidophilus*, *B. breve*, and *B. infantis*, and their synbiotic mixtures can downregulate IL-8 production by the TNF-α stimulated HT-29 cells induced by an NEC-positive associated pathogenic bacterium, *K. oxytoca*. The digestion of Tc-WS-NSP, the type of probiotic bacteria, the probiotic bacterial concentration, and the composition of the synbiotic mixture influenced the ability of the Tc-WS-NSP, the probiotic, and the synbiotic mixture to downregulate the IL-8 production. The combination of Tc-WS-NSP with the probiotic can offer higher beneficial effect than the use of either Tc-WS-NSP or each of the probiotics solely. The ability demonstrated by the synbiotic mixture of the digested Tc-WS-NSP-ICE and high bacterial concentration of *B. breve*, to downregulate the IL-8 produced by the TNF-α stimulated HT-29 cells in the presence of an NEC-positive associated pathogen, *K. oxytoca* is a novel and a significant finding that may be useful in the prevention of NEC. The use of synbiotics can be considered as an “infant friendly” approach in the prevention of, as well as a nutritional intervention for, NEC. With further and more in-depth research studies to support this finding and validate its efficiency, the use of this approach could minimize, if not completely prevent, the need for infants to undergo complex medical interventions such as the use of antibiotics and surgery to treat NEC. This infant-friendly approach could potentially avert the known long-term side effects of the use of antibiotics and surgery that an infant may suffer during its growth and development. On a larger scale, the synbiotic mixtures may also be useful for other gastrointestinal diseases associated with the unregulated production of pro-inflammatory cytokines caused by the presence of *K. oxytoca*.

## Figures and Tables

**Table 1:** IL-8 reduction (%) upon incubation of undigested or digested Tc-WS-NSP extracted using the conventional extraction (CE) and improved conventional extraction (ICE) methods in the presence of non-pathogenic *E.coli* or NEC-positive associated pathogen *K. oxytoca*.

Bacterial Isolate	Bacterial Isolate Concentration (CFU/mL)	Tc-WS-NSP		IL-8 Reduction (%)
*K. oxytoca*	4.0 × 10^6^	CE	Undigested	17.2 ± 3.0 ^BC,M^
			Digested	21.3 ± 2.4 ^AB,LM^
		ICE	Undigested	19.2 ± 0.8 ^AB,LM^
			Digested	24.5 ± 3.4 ^A,L^
	2.1 × 10^7^	CE	Undigested	18.1 ± 1.2 ^ABC,M^
			Digested	21.8 ± 1.0 ^AB,LM^
		ICE	Undigested	16.8 ± 2.0 ^BC,M^
			Digested	20.5 ± 2.3 ^AB,LM^
*E. coli*	4.3 × 10^6^	CE	Undigested	8.4 ± 3.0 ^DE,MN^
			Digested	10.0 ± 1.3 ^DE,LM^
		ICE	Undigested	5.2 ± 2.0 ^E,N^
			Digested	12.2 ± 0.8 ^CD,MN^
	1.3 × 10^7^	CE	Undigested	8.1 ± 2.4 ^DE,MN^
			Digested	16.6 ± 2.6 ^BC,L^
		ICE	Undigested	8.1 ± 0.7 ^DE,MN^
			Digested	17.1 ± 2.7 ^BC,L^

Values are mean ± SD (*n =* 3). Means that do not share the same letters are significantly (*p ≤* 0.05) different (ANOVA: General Linear Model with Tukey pairwise comparison). Grouping information on statistical differences: A–E, among treatments; L–N, between treatments of different bacterial isolate (*K. oxytoca* or *E. coli*).

**Table 2:** IL-8 reduction (%) upon incubation with each of the probiotics *L. acidophilus*, *B. breve*, and *B. infantis* in the presence of non-pathogenic *E. coli* or NEC-positive associated pathogen *K. oxytoca*.

Bacterial Isolate		*L. acidophilus*	IL-8 Reduction (%)	*B. breve*	IL-8 Reduction (%)	*B. infantis*	IL-8 Reduction (%)
*K. oxytoca*	4.0 × 10^6^	3.1 × 10^6^	29.1±2.2 ^H,NO,X^	3.73 × 10^5^	31.7 ± 1.0 ^EFGH,N,Y^	1.53 × 10^5^	29.5 ± 1.0 ^GH,N,X^
	2.1 × 10^7^		28.5 ± 2.5 ^H,O,X^		31.7 ± 1.3 ^EFGH,N,Y^		30.2 ± 1.0 ^FGH,MN,X^
	4.0 × 10^6^	5.6 × 10^9^	32.3 ± 1.5 ^EFGH,MNO,X^	2.70 × 10^8^	36.3 ± 2.7 ^CDEFGH,N,XY^	4.56 × 10^8^	32.0 ± 2.7 ^EFGH,LMN,X^
	2.1 × 10^7^		31.2 ± 2.9 ^EFGH,MNO,X^		37.8 ± 1.9 ^BCDEFGH,MN,X^		33.1 ± 2.9 ^DEFGH,LMN,X^
*E. coli*	4.3 × 10^6^	3.1 × 10^6^	37.5 ± 2.5 ^BCDEFGH,LMN,X^	3.73 × 10^5^	45.9 ± 4.1 ^ABC,LM,X^	1.53 × 10^5^	41.2 ± 3.9 ^ABCDEF,LMN,X^
	1.3 × 10^7^		35.9 ± 5.1 ^CDEFGH,LMNO,X^		46.7 ± 5.2 ^ABC,LM,X^		42.5 ± 4.8 ^ABCDE,LM,X^
	4.3 × 10^6^	5.6 × 10^9^	39.6 ± 2.8 ^ABCDEFGH,LM,X^	2.70 × 10^8^	48.4 ± 4.0 ^AB,L,X^	4.56 × 10^8^	44.1 ± 7.5 ^ABCD,L,X^
	1.3 × 10^7^		40.9 ± 3.3 ^ABCDEFG,L,X^		50.2 ± 3.3 ^A,L,X^		44.3 ± 7.3 ^ABCD,L,X^

Values are mean ± SD (*n =* 3). Means that do not share the same letters are significantly (*p* *≤* 0.05) different (ANOVA and General linear model using Tukey pairwise comparison). Grouping information on statistical difference: A–H, among treatments, L–O, between treatments for each of the probiotics, X–Y, between bacterial isolate (*K. oxytoca* or *E. coli*) for each probiotic.

**Table 3:** IL-8 reduction (%) upon incubation with undigested or digested Tc-WS-NSP extracted using the conventional extraction (CE) and improved conventional extraction (ICE) methods and *L. acidophilus* in the presence of non-pathogenic *E. coli* or NEC-positive associated pathogen *K. oxytoca*.

T WS-NSP		*L. acidophilus* (CFU/mL)	*E. coli* (CFU/mL)	IL-8 Reduction (%)	*K. oxytoca* (CFU/mL)	IL-8 Reduction (%)
CE	Undigested	3.08 × 10^6^	4.33 × 10^6^	43.0 ± 1.8 ^ABCDE,L,Q,X^	4.00 × 10^6^	32.5 ± 3.8 ^E,L,Q,X^
	Digested			43.8 ± 1.4 ^ABCDE,L,Q,X^		33.4 ± 5.4 ^CDE,L,Q,X^
ICE	Undigested			41.8 ± 4.4 ^ABCDE,L,Q,X^		32.5 ± 4.1 ^E,L,Q,X^
	Digested			43.6 ± 2.4 ^ABCDE,L,Q,X^		33.2 ± 4.1 ^CDE,L,Q,X^
CE	Undigested	5.57 × 10^9^		44.4 ± 2.2 ^ABCD,L,Q,X^		38.5 ± 3.8 ^ABCDE,L,Q,X^
	Digested			45.0 ± 3.7 ^AB,L,Q,X^		40.3 ± 2.8 ^ABCDE,L,Q,X^
ICE	Undigested			44.5 ± 3.5 ^ABC,L,Q,X^		40.8 ± 1.9 ^ABCDE,L,Q,X^
	Digested			46.3 ± 4.3 ^A,L,Q,X^		41.3 ± 2.5 ^ABCDE,L,Q,X^
CE	Undigested	3.08 × 10^6^	1.33 × 10^7^	40.5 ± 1.7 ^ABCDE,L,Q,X^	2.05 × 10^7^	33.0 ± 4.7 ^E,L,Q,X^
	Digested			43.7 ± 1.2 ^ABCDE,L,Q,X^		34.8 ± 6.4 ^BCDE,L,Q,X^
ICE	Undigested			42.8 ± 4.2 ^ABCDE,L,Q,X^		33.1 ± 4.4 ^DE,L,Q,X^
	Digested			43.7 ± 3.1 ^ABCDE,L,Q,X^		34.9 ± 5.3 ^BCDE,L,Q,X^
CE	Undigested	5.57 × 10^9^		43.2 ± 4.0 ^ABCDE,L,Q,X^		40.4 ± 2.7 ^ABCDE,L,Q,X^
	Digested			45.7 ± 2.5 ^AB,L,Q,X^		40.8 ± 2.1 ^ABCDE,L,Q,X^
ICE	Undigested			45.6 ± 2.4 ^AB,L,Q,X^		41.2 ± 3.3 ^ABCDE,L,Q,X^
	Digested			46.8 ± 2.9 ^A,L,Q,X^		42.0 ± 2.5 ^ABCDE,L,Q,X^

Values are mean ± SD (*n =* 3). Means that do not share the same letters are significantly (*p* ≤ 0.05) different (ANOVA and General linear model using Tukey pairwise comparison). Grouping information on statistical difference: A–E, IL-8 reduction (%) among all treatments; L, IL-8 reduction (%) between bacterial isolate (*E. coli* or *K. oxytoca*); Q, IL-8 reduction (%) between treatments at different bacterial concentrations of *E. coli* or *K. oxytoca*; X, IL-8 reduction (%) between treatments at different bacterial concentrations of *L acidophilus.*

**Table 4:** IL-8 reduction (%) upon incubation with undigested or digested Tc-WS-NSP extracted using the conventional extraction (CE) and improved conventional extraction (ICE) methods and *B. breve* in the presence of non-pathogenic *E. coli* or NEC-positive associated pathogen *K. oxytoca*.

T WS-NSP		*B. breve* (CFU/mL)	*E. coli* (CFU/mL)	IL-8 Reduction (%)	*K. oxytoca* (CFU/mL)	IL-8 Reduction (%)
CE	Undigested	3.7 × 10^5^	4.3 × 10^6^	51.7 ± 3.7 ^ABC,L,Q,X^	4.0 × 10^6^	38.2 ± 2.7 ^E,L,Q,X^
	Digested			51.5 ± 1.9 ^ABCD,L,Q,X^		39.3 ± 2.6 ^E,L,Q,X^
ICE	Undigested			51.4 ± 2.6 ^ABCD,L,Q,X^		38.6 ± 4.1 ^E,L,Q,X^
	Digested			52.3 ± 3.1 ^AB,L,Q,X^		39.2 ± 4.1 ^E,L,Q,X^
CE	Undigested	2.7 × 10^8^		54.4 ± 2.6 ^A,L,Q,X^		40.3 ± 3.3 ^E,L,Q,X^
	Digested			55.2 ± 3.0 ^A,L,Q,X^		41.5 ± 2.3 ^E,L,Q,X^
ICE	Undigested			54.5 ± 2.1 ^A,L,Q,X^		42.0 ± 1.9 ^E,L,Q,X^
	Digested			55.2 ± 2.3 ^A,L,Q,X^		43.2 ± 2.5 ^DE,L,Q,X^
CE	Undigested	3.7 × 10^5^	1.3 × 10^7^	54.0 ± 2.1 ^A,L,Q,X^	2.1 × 10^7^	41.4 ± 4.1 ^E,L,Q,X^
	Digested			54.4 ± 2.4 ^A,L,Q,X^		41.9 ± 5.9 ^E,L,Q,X^
ICE	Undigested			54.3 ± 2.4 ^A,L,Q,X^		41.4 ± 3.8 ^E,L,Q,X^
	Digested			55.0 ± 1.9 ^A,L,Q,X^		42.0 ± 4.7 ^E,L,Q,X^
CE	Undigested	2.7 × 10^8^		56.0 ± 2.1 ^A,L,Q,X^		43.3 ± 2.1 ^CDE,L,Q,X^
	Digested			57.1 ± 2.4 ^A,L,Q,X^		44.2 ± 1.6 ^BCDE,L,Q,X^
ICE	Undigested			56.1 ± 2.6 ^A,L,Q,X^		43.7 ± 3.3 ^CDE,L,Q,X^
	Digested			58.1 ± 2.3 ^A,L,Q,X^		45.0 ± 2.5 ^BCDE,L,Q,X^

Values are mean ± SD (*n =* 3). Means that do not share the same letters are significantly (*p* *≤* 0.05) different (ANOVA and General linear model using Tukey pairwise comparison). Grouping information on statistical difference: A–E, IL-8 reduction (%) among all treatments; L, IL-8 reduction (%) between bacterial isolate (*E. coli* or *K. oxytoca*); Q, IL-8 reduction (%) between treatments at different bacterial concentrations of *E. coli* or *K. oxytoca*; X, IL-8 reduction (%) between treatments at different bacterial concentrations of *B. breve.*

**Table 5:** IL-8 reduction (%) upon incubation with undigested and digested T WS-NSP extracted using the conventional extraction (CE) and improved conventional extraction (ICE) methods and *B. infantis* in the presence of non-pathogenic *E. coli* or NEC-positive associated pathogen *K. oxytoca*.

T WS-NSP		*B. infantis* (CFU/mL)	*E. coli* (CFU/mL)	IL-8 Reduction (%)	*K. oxytoca* (CFU/mL)	IL-8 Reduction (%)
CE	Undigested	4.56 × 10^5^	4.33 × 10^6^	49.2 ± 3.1 ^ABCDEF,L,Q,X^	4.00 × 10^6^	34.1 ± 2.3 ^G,L,S,X^
	Digested			50.6 ± 2.9 ^ABCD,L,Q,X^		35.4 ± 1.3 ^G,L,RS,X^
ICE	Undigested			49.3 ± 2.2 ^ABCDEF,L,Q,X^		35.2 ± 1.5 ^G,L,RS,X^
	Digested			51.4 ± 3.4 ^ABC,L,Q,X^		35.5 ± 1.7 ^G,L,RS,X^
CE	Undigested	1.53 × 10^8^		52.0 ± 4.1 ^ABC,L,Q,X^		38.3 ± 1.4 ^G,L,QRS,X^
	Digested			52.4 ± 3.0 ^AB,L,Q,X^		38.9 ± 1.1 ^G,L,QR,X^
ICE	Undigested			52.4 ± 4.5 ^AB,L,Q,X^		39.4 ± 1.1 ^FG,L,QR,X^
	Digested			54.2 ± 3.6 ^A,L,Q,X^		40.9 ± 2.1 ^DEFG,L,Q,X^
CE	Undigested	4.56 × 10^5^	1.33 × 10^7^	50.7 ± 1.3 ^ABCD,L,Q,X^	2.05 × 10^7^	37.8 ± 1.9 ^G,L,Q,X^
	Digested			52.5 ± 2.9 ^AB,L,Q,X^		39.7 ± 5.7 ^FG,L,Q,X^
ICE	Undigested			50.4 ± 2.1 ^ABCDE,L,Q,X^		39.7 ± 5.1 ^FG,L,Q,X^
	Digested			52.1 ± 0.7 ^ABC,L,Q,X^		40.3 ± 4.8 ^EFG,L,Q,X^
CE	Undigested	1.53 × 10^8^		52.4 ± 2.8 ^AB,L,Q,X^		41.1 ± 3.4 ^DEFG,L,Q,X^
	Digested			53.1 ± 3.1 ^AB,L,Q,X^		42.2 ± 4.6 ^CDEFG,L,Q,X^
ICE	Undigested			52.1 ± 1.2 ^ABC,L,Q,X^		42.1 ± 4.5 ^CDEFG,L,Q,X^
	Digested			53.7 ± 2.3 ^A,L,Q,X^		43.1 ± 5.1 ^BCDEFG,L,Q,X^

Values are Mean ± SD (*n =* 3). Means that do not share a letter are significantly (*p ≤* 0.05) different (ANOVA and General linear model using Tukey pairwise comparison). Grouping information on statistical difference: A–G, IL-8 reduction (%) among all treatments; L, IL-8 reduction (%) between bacterial isolate (*E. coli* or *K. oxytoca*); Q–S, IL-8 reduction (%) between treatments at different bacterial concentrations of *E. coli* or *K. oxytoca*; X, IL-8 reduction (%) between treatments at different bacterial concentrations of *B. infantis.*

## Data Availability

Not applicable.

## Supplementary Materials

The following are available online at https://www.mdpi.com/article/10.3390/nu14102128/s1, Figure S1: Viable TNF-α-stimulated HT-29 cells (%) incubated with undigested and digested Tc-WS-NSP extracted using the CE and ICE methods, *K. oxytoca* or *E. coli* at different bacterial concentrations (CFU/mL). Bars that do not share the same letters are significantly (*p ≤* 0.05) different (ANOVA with Tukey pairwise comparison). Grouping information for significant differences: A–C, viable cells among treatments compared to control samples, Figure S2: Viable TNF-α-stimulated HT-29 cells (%) incubated with *L. acidophilus*, *K. oxytoca* or *E. coli* at different bacterial concentrations (CFU/mL). Bars that do not share the same letter are significantly (*p ≤* 0.05) different (ANOVA with Tukey pairwise comparison). Grouping information for significant differences: A-B, viable cells among treatments compared to control samples, Figure S3: Viable TNF-α-stimulated HT-29 cells (%) incubated with undigested and digested Tc-WS-NSP (CE and ICE), *L. acidophilus*, *K. oxytoca* or *E. coli* at different bacterial concentrations (CFU/mL). Bars that do not share the same letter are significantly (*p ≤* 0.05) different (ANOVA with Tukey pairwise comparison). Grouping information for significant differences: A-B, viable cells among treatments compared to control samples, Figure S4: Viable TNF-α-stimulated HT-29 cells (%) incubated with undigested and digested Tc-WS-NSP (CE and ICE), *B. breve*, *K. oxytoca* or *E. coli* at different bacterial concentrations (CFU/mL). Bars that do not share the same letter are significantly (*p ≤* 0.05) different (ANOVA with Tukey pairwise comparison). Grouping information for significant differences: A-B, viable cells among treatments compared to control samples, Figure S5: Viable TNF-α-stimulated HT-29 cells (%) incubated with undigested and digested Tc-WS-NSP (CE and ICE), *B. infantis*, *K. oxytoca* or *E. coli* at different bacterial concentrations (CFU/mL). Bars that do not share the same letter are significantly (*p ≤* 0.05) different (ANOVA with Tukey pairwise comparison). Grouping information for significant differences: A-B, viable cells among treatments compared to control samples, Figure S6: IL-8 production by TNF-α-stimulated HT-29 cells incubated with undigested or digested Tc-WS-NSP extracted using the CE and ICE methods with heat-killed *E. coli*/*K. oxytoca* at different bacterial concentrations (CFU/mL). Values are mean ± SD (*n =* 3) of the IL-8 reduction (%) upon incubation of undigested or digested Tc-WS-NSP-CE or Tc-WS-NSP-ICE. Means that do not share the same letters are significantly (*p ≤* 0.05) different (ANOVA with Tukey pairwise comparison). Grouping information on statistical differences: A-E, among treatments; L-N, between treatments of different bacterial isolates (*K. oxytoca* or *E. coli*), Figure S7: IL-8 production by TNF-α-stimulated HT-29 cells incubated with probiotics with heat-killed *E. coli*/*K. oxytoca* at different bacterial concentrations (CFU/mL). Values are mean ± SD (*n =* 3) of the IL-8 reduction (%) upon incubation of the probiotics *L. acidophilus*, *B. breve*, and *B. infantis*. Means that do not share the same letters are significantly (*p ≤* 0.05) different (ANOVA with Tukey pairwise comparison). Grouping information on statistical difference: A-H, among treatments, L-O, between treatments for each of the probiotics, X-Y, between bacterial isolate (*K. oxytoca* or *E. coli*) for each probiotic, Figure S8: IL-8 production by TNF-α-stimulated HT-29 cells incubated with undigested or digested Tc-WS-NSP extracted using the CE and ICE methods and *L. acidophilus* with heat-killed *E. coli*/*K. oxytoca* at different bacterial concentrations (CFU/mL). Values are mean ± SD (*n =* 3) of the IL-8 reduction (%) upon incubation of the undigested or digested Tc-WS-NSPs and the probiotic *L. acidophilus*. Means that do not share the same letters are significantly (*p ≤* 0.05) different (ANOVA with Tukey pairwise comparison). Grouping information on statistical difference: A-E, IL-8 reduction (%) among all treatments; L, IL-8 reduction (%) between bacterial isolate (*E. coli* or *K. oxytoca*); Q-S, IL-8 reduction (%) between treatments at different bacterial concentrations of *E. coli* or *K. oxytoca*; X, IL-8 reduction (%) between treatments at different bacterial concentrations of *L acidophilus*, Figure S9: IL-8 production by TNF-α-stimulated HT-29 cells incubated with undigested or digested Tc-WS-NSP extracted using the CE and ICE methods and *B. breve* with heat-killed *E. coli*/*K. oxytoca* at different bacterial concentrations (CFU/mL). Values are mean ± SD (*n =* 3) of the IL-8 reduction (%) upon incubation of the undigested or digested Tc-WS-NSPs and the probiotic *B. breve*. Means that do not share the same letters are significantly (*p ≤* 0.05) different (ANOVA with Tukey pairwise comparison). Grouping information on statistical difference: A-E, IL-8 reduction (%) among all treatments; L, IL-8 reduction (%) between bacterial isolate (*E. coli or K. oxytoca*); Q-S, IL-8 reduction (%) between treatments at different bacterial concentrations of *E. coli or K. oxytoca*; X, IL-8 reduction (%) between treatments at different bacterial concentrations of *B. breve*, Figure S10: IL-8 production by TNF-α-stimulated HT-29 cells incubated with undigested or digested Tc-WS-NSP extracted using the CE and ICE methods and *B. infantis* with heat-killed *E. coli*/*K. oxytoca* at different bacterial concentrations (CFU/mL). Values are mean ± SD (*n =* 3) of the IL-8 reduction (%) upon incubation of the undigested or digested Tc-WS-NSPs and the probiotic *B. infantis*. Means that do not share the same letters are significantly (*p ≤* 0.05) different (ANOVA with Tukey pairwise comparison). Grouping information on statistical difference: A-E, IL-8 reduction (%) among all treatments; L, IL-8 reduction (%) between bacterial isolate (*E. coli or K. oxytoca*); Q-S, IL-8 reduction (%) between treatments at different bacterial concentrations of *E. coli* or *K. oxytoca*; X, IL-8 reduction (%) between treatments at different bacterial concentrations of *B. infantis.*
