# Supplementary material for: Effects of Taro (Colocasia esculenta) Water-Soluble Non-Starch Polysaccharide, Lactobacillus acidophilus, Bifidobacterium breve, Bifidobacterium infantis, and Their Synbiotic Mixtures on Pro-Inflammatory Cytokine Interleukin-8 Production
Source: Nutrients. 2022 May 19;14(10):2128. doi: 10.3390/nu14102128 (PMC9147535; doi:10.3390/nu14102128)
Supplement: Supplementary file 1 [file nutrients-14-02128-s001.zip › nutrients-1637292-supplementary.pdf]

Supplementary Materials:

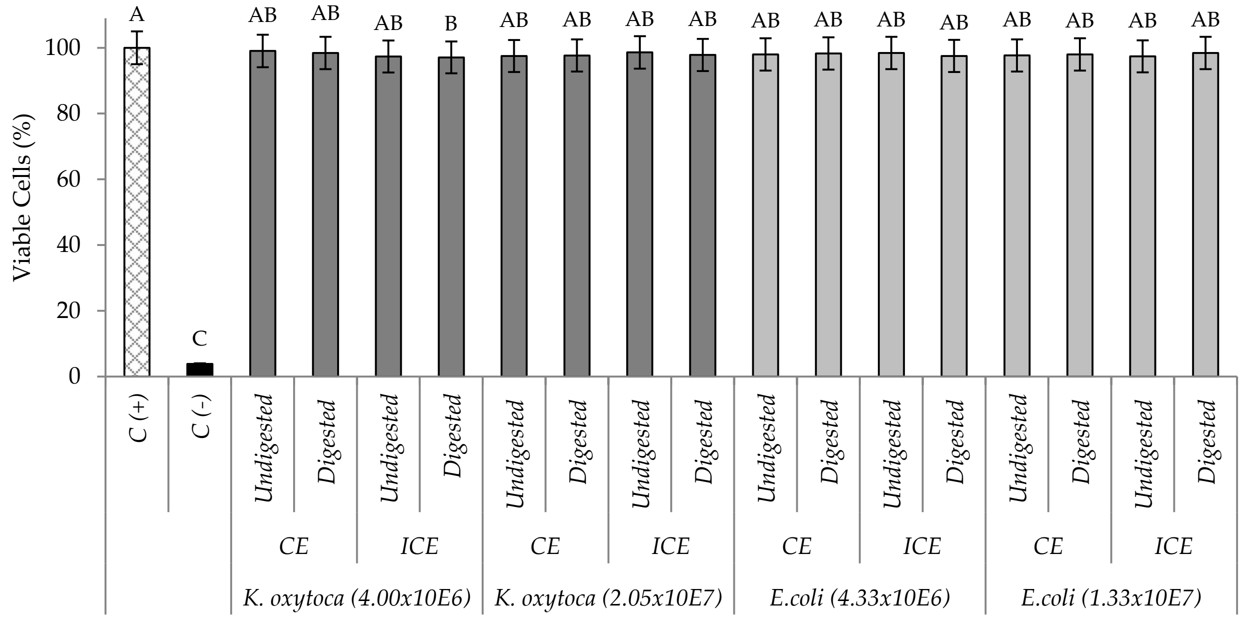

Supplemental Figure S1. Viable TNF-α-stimulated HT-2 9 cells (%) incubated with undigested and digested Tc-WS-NSP extracted using the CE and ICE methods, *K. oxytoca* or *E. coli* at different bacterial concentrations (CFU/mL). Bars that do not share the same letters are significantly ($p \leq 0.05$) different (ANOVA with Tukey pairwise comparison). Grouping information for significant differences: A–C, viable cells among treatments compared to control samples.

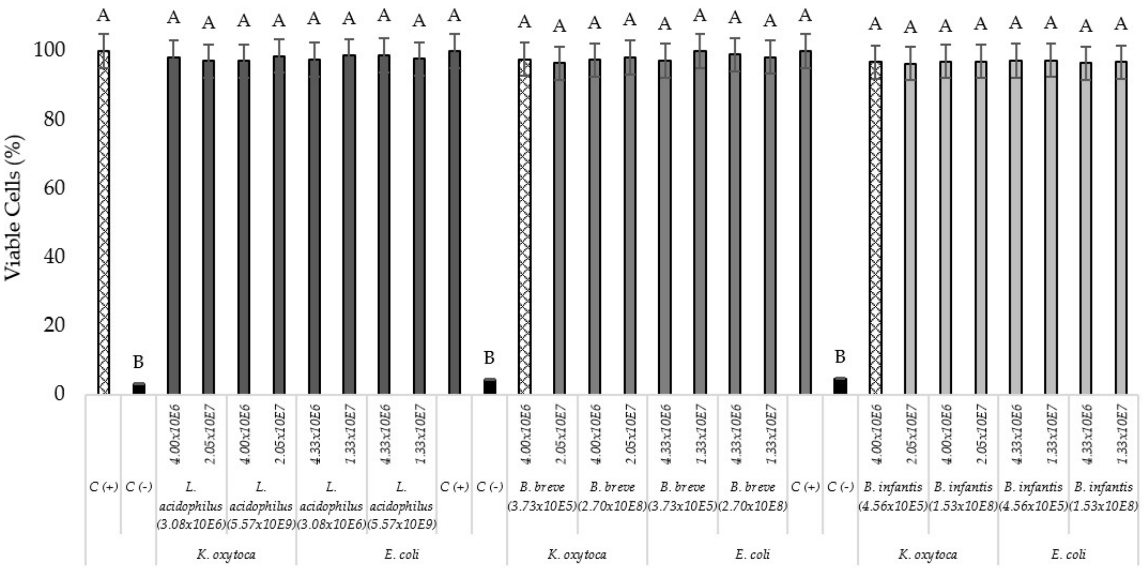

Supplemental Figure S2. Viable TNF-α-stimulated HT-29 cells (%) incubated with *L. acidophilus*, *K. oxytoca* or *E. coli* at different bacterial concentrations (CFU/mL). Bars that do not share the same letter are significantly ($p \leq 0.05$) different (ANOVA with Tukey pairwise comparison). Grouping information for significant differences: A-B, viable cells among treatments compared to control samples.

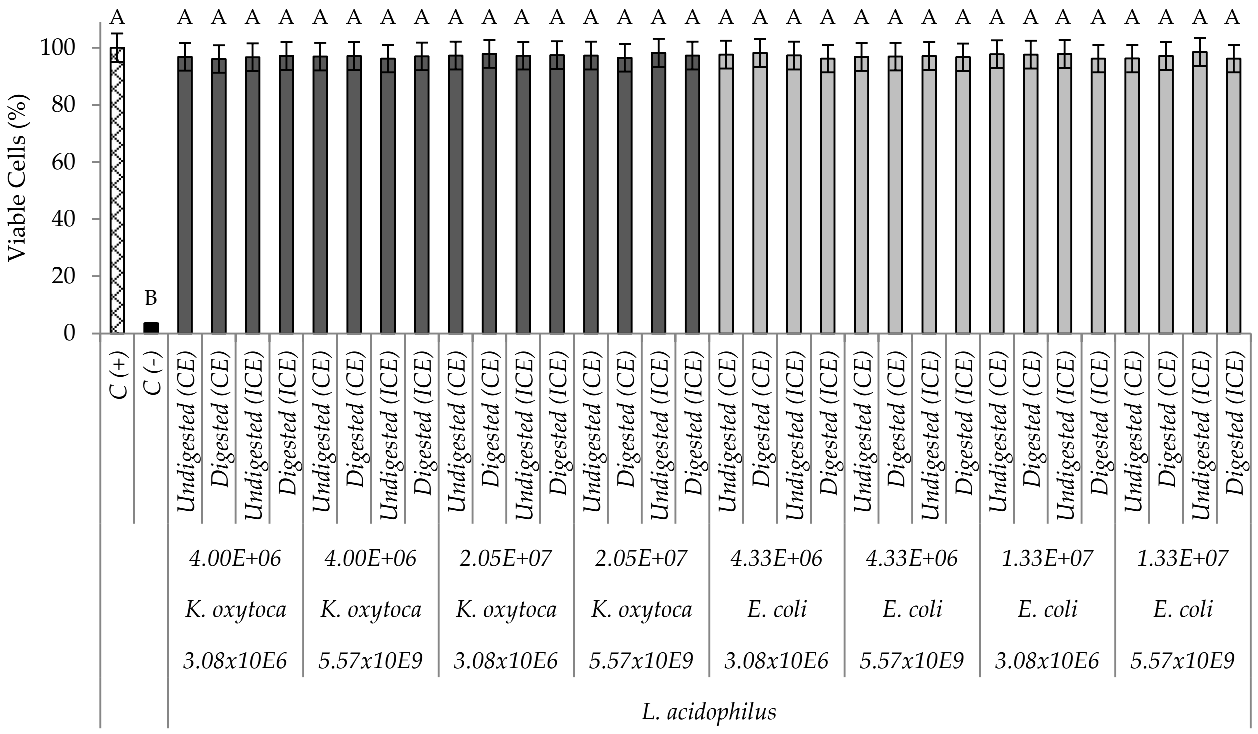

Supplemental Figure S3. Viable TNF-α-stimulated HT-29 cells (%) incubated with undigested and digested Tc-WS-NSP (CE and ICE), *L. acidophilus*, *K. oxytoca* or *E. coli* at different bacterial concentrations (CFU/mL). Bars that do not share the same letter are significantly ($p \leq 0.05$) different (ANOVA with Tukey pairwise comparison). Grouping information for significant differences: A-B, viable cells among treatments compared to control samples.

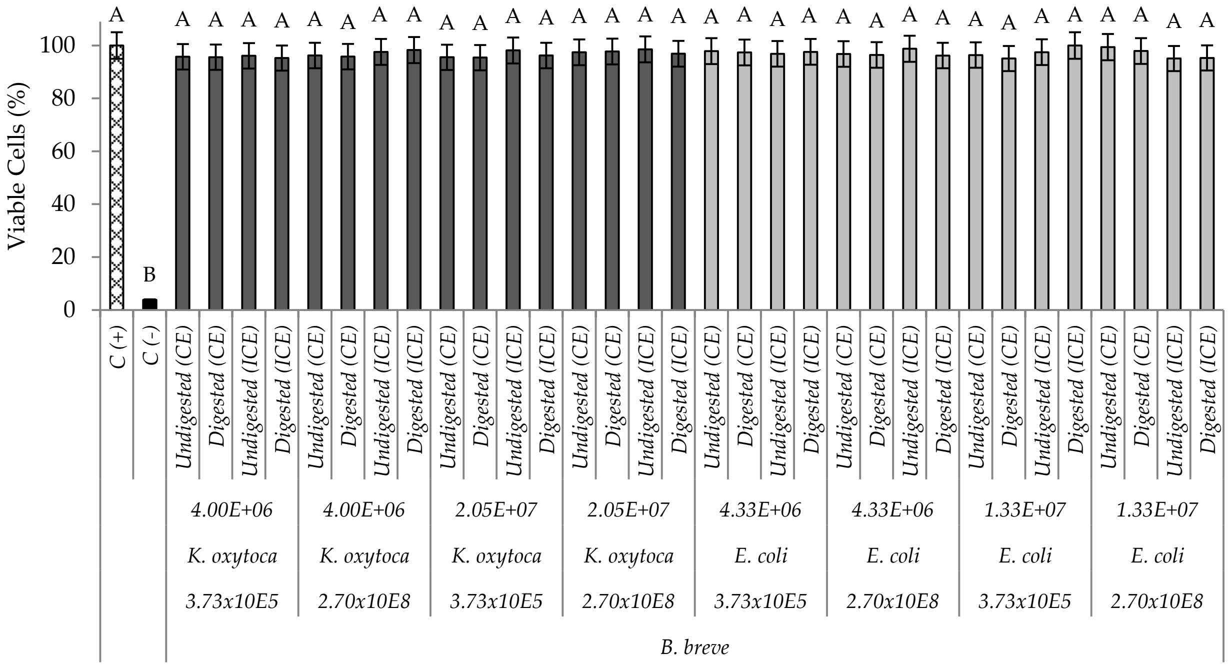

Supplemental Figure S4. Viable TNF-α-stimulated HT-29 cells (%) incubated with undigested and digested Tc-WS-NSP (CE and ICE), *B. breve*, *K. oxytoca* or *E. coli* at different bacterial concentrations (CFU/mL). Bars that do not share the same letter are significantly ($p \leq 0.05$) different (ANOVA with Tukey pairwise comparison). Grouping information for significant differences: A-B, viable cells among treatments compared to control samples.

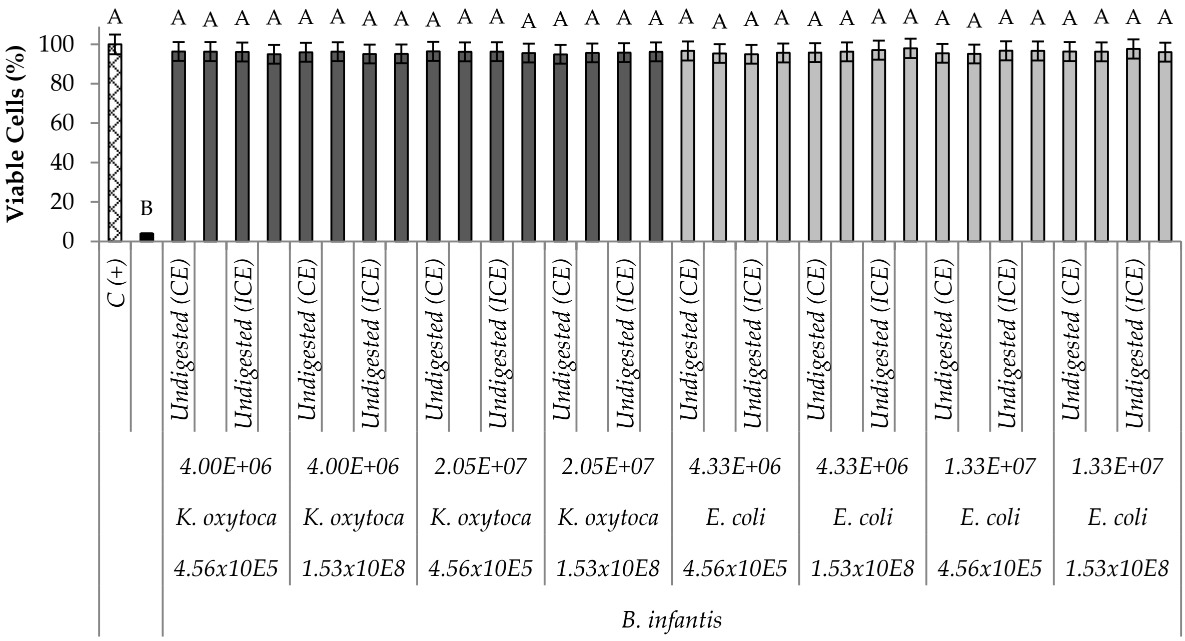

Supplemental Figure S5. Viable TNF-α-stimulated HT-29 cells (%) incubated with undigested and digested Tc-WS-NSP (CE and ICE), *B. infantis*, *K. oxytoca* or *E. coli* at different bacterial concentrations (CFU/mL). Bars that do not share the same letter are significantly ($p \leq 0.05$) different (ANOVA with Tukey pairwise comparison). Grouping information for significant differences: A-B, viable cells among treatments compared to control samples.

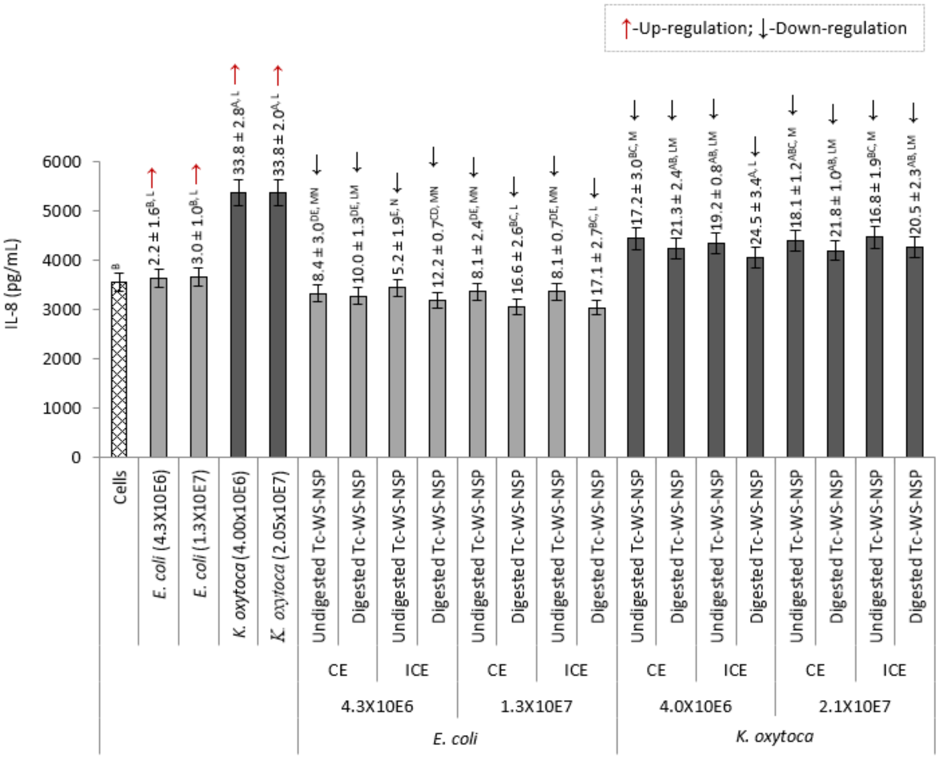

Supplemental Figure S6. IL-8 production by TNF-α-stimulated HT-29 cells incubated with undigested or digested Tc-WS-NSP extracted using the CE and ICE methods with heat-killed *E. coli*/ *K. oxytoca* at different bacterial concentrations (CFU/mL). Values are mean ± SD (n = 3) of the IL-8 reduction (%) upon incubation of undigested or digested Tc-WS-NSP-CE or Tc-WS-NSP-ICE. Means that do not share the same letters are significantly ($p \leq 0.05$) different (ANOVA and General Linear Model with Tukey pairwise comparison). Grouping information on statistical differences: A-E, among treatments; L-N, between treatments of different bacterial isolate (*K. oxytoca* or *E. coli*).

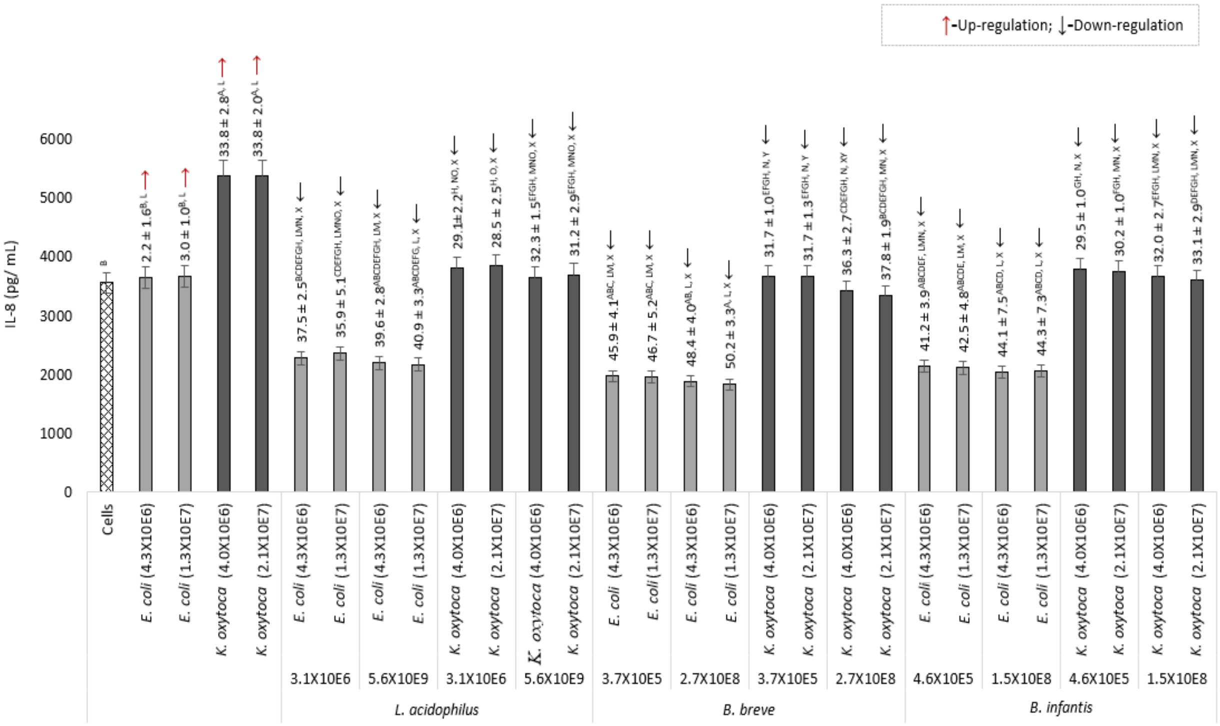

Supplemental Figure S7. IL-8 production by TNF-α-stimulated HT-29 cells incubated with probiotics with heat-killed E. coli/ K. oxytoca at different bacterial concentrations (CFU/mL). Values are mean ± SD (n = 3) of the IL-8 reduction (%) upon incubation of the probiotics *L. acidophilus*, *B. breve*, and *B. infantis*. Means that do not share the same letters are significantly ($p \leq 0.05$) different (ANOVA and General Linear Model with Tukey pairwise comparison). Grouping information on statistical difference: A-H, among treatments, L-O, between treatments for each of the probiotics, X-Y, between bacterial isolate (*K. oxytoca* or *E. coli*) for each probiotic.

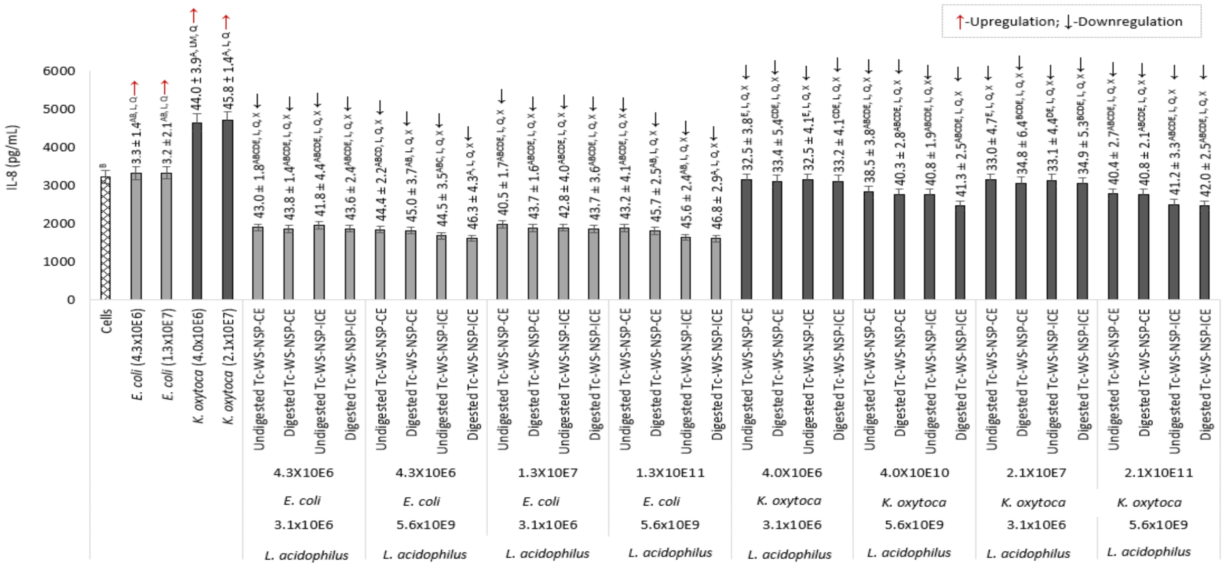

Supplemental Figure S8. IL-8 production by TNF-α-stimulated HT-29 cells incubated with undigested or digested Tc-WS-NSP extracted using the CE and ICE methods and *L. acidophilus* with heat-killed *E. coli/ K. oxytoca* at different bacterial concentrations (CFU/mL). Values are mean ± SD (n = 3) of the IL-8 reduction (%) upon incubation of the undigested or digested Tc-WS-NSPs and the probiotic *L. acidophilus*. Means that do not share the same letters are significantly ($p \leq 0.05$) different (ANOVA and General Linear Model with Tukey pairwise comparison). Grouping information on statistical difference: A-E, IL-8 reduction (%) among all treatments; L, IL-8 reduction (%) between bacterial isolate (*E. coli* or *K. oxytoca*); Q-S, IL-8 reduction (%) between treatments at different bacterial concentrations of *E. coli* or *K. oxytoca*; X, IL-8 reduction (%) between treatments at different bacterial concentrations of *L acidophilus*.

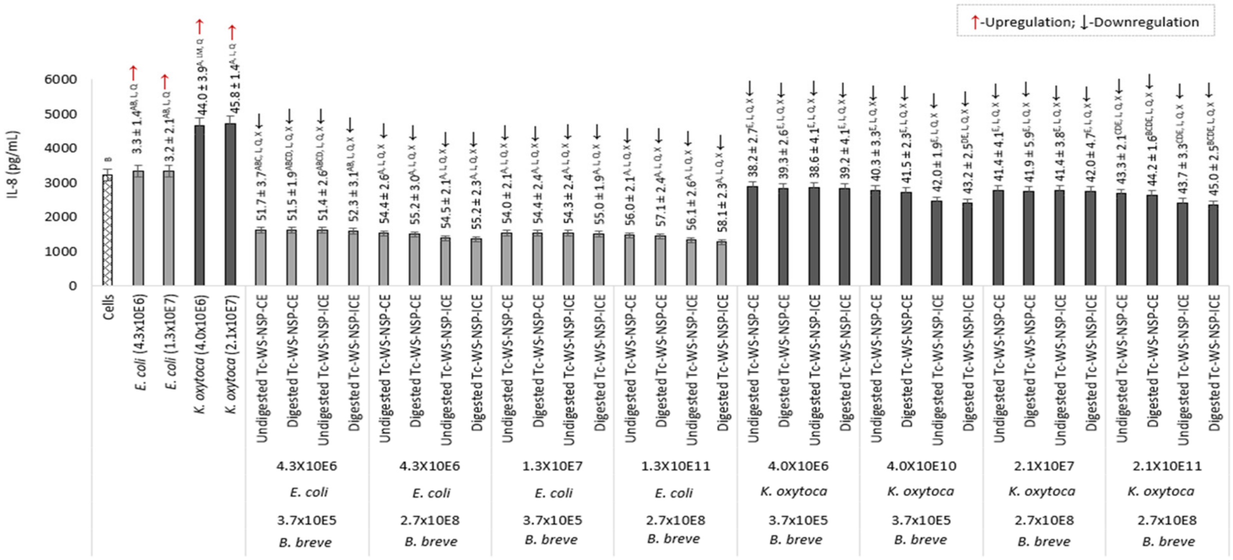

Supplemental Figure S9. IL-8 production by TNF-α-stimulated HT-29 cells incubated with undigested or digested Tc-WS-NSP extracted using the CE and ICE methods and *B. breve* with heat-killed *E. coli*/ *K. oxytoca* at different bacterial concentrations (CFU/mL). Values are mean ± SD ($n = 3$) of the IL-8 reduction (%) upon incubation of the undigested or digested Tc-WS-NSPs and the probiotic *B. breve*. Means that do not share the same letters are significantly ($p \leq 0.05$) different (ANOVA and General Linear Model with Tukey pairwise comparison). Grouping information on statistical difference: A-E, IL-8 reduction (%) among all treatments; L, IL-8 reduction (%) between bacterial isolate (*E. coli or K. oxytoca*); Q-S, IL-8 reduction (%) between treatments at different bacterial concentrations of *E. coli or K. oxytoca*; X, IL-8 reduction (%) between treatments at different bacterial concentrations of *B. breve.*

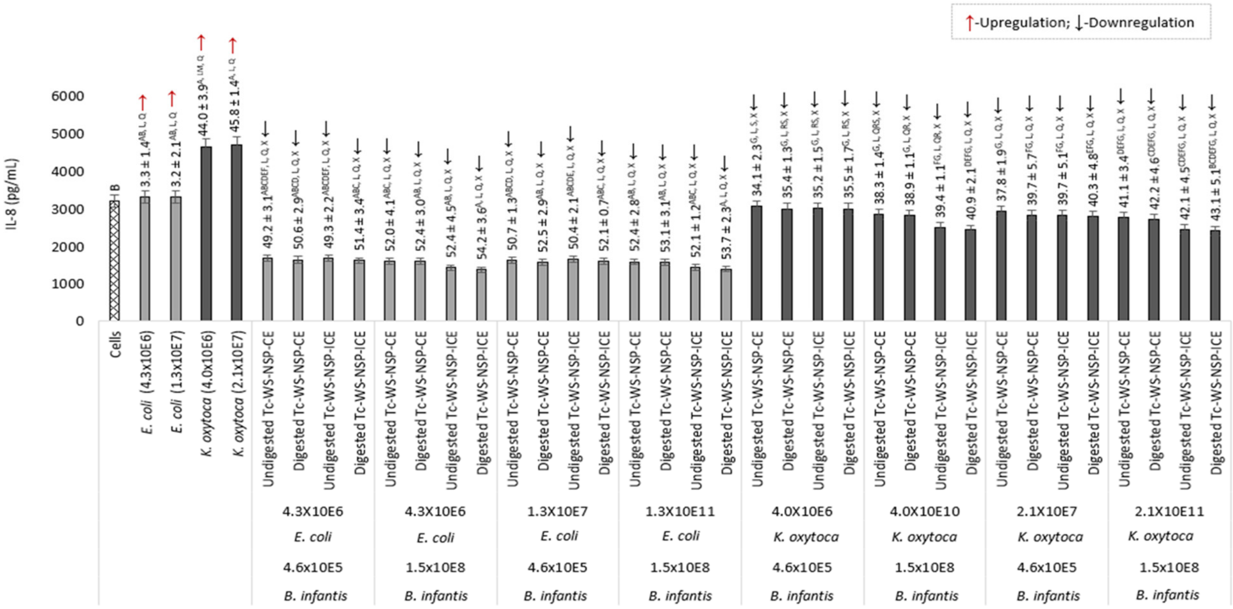

Supplemental Figure S10. IL-8 production by TNF-α-stimulated HT-29 cells incubated with undigested or digested Tc-WS-NSP extracted using the CE and ICE methods and *B. infantis* with heat-killed *E. coli*/ *K. oxytoca* at different bacterial concentrations (CFU/mL). Values are mean ± SD ($n = 3$) of the IL-8 reduction (%) upon incubation of the undigested or digested Tc-WS-NSPs and the probiotic *B. infantis*. Means that do not share the same letters are significantly ($p \leq 0.05$) different (ANOVA and General Linear Model with Tukey pairwise comparison). Grouping information on statistical difference: A-E, IL-8 reduction (%) among all treatments; L, IL-8 reduction (%) between bacterial isolate (*E. coli or K. oxytoca*); Q-S, IL-8 reduction (%) between treatments at different bacterial concentrations of *E. coli* or *K. oxytoca*; X, IL-8 reduction (%) between treatments at different bacterial concentrations of *B. infantis.*
